# The use of micelles to deliver potential hedgehog pathway inhibitor for the treatment of liver fibrosis

**DOI:** 10.7150/thno.38913

**Published:** 2019-10-12

**Authors:** Virender Kumar, Yuxiang Dong, Vinod Kumar, Saud Almawash, Ram I. Mahato

**Affiliations:** Department of Pharmaceutical Sciences, University of Nebraska Medical Center, Omaha, NE 68198, USA

**Keywords:** Liver fibrosis, Hedgehog, MDB5, GDC-0449.

## Abstract

**Rationale:** Hedgehog (Hh) pathway plays an essential role in liver fibrosis by promoting the proliferation of hepatic stellate cells (HSCs) by enhancing their metabolism via yes-associated protein 1 (YAP1). Despite the presence of several inhibitors, Hh signaling cannot be controlled exclusively due to their poor efficacy and the lack of a suitable delivery system to the injury site. Therefore, it is rationale to develop new potent Hh inhibitors and suitable delivery carriers.

**Methods:** Based on the structure and activity of Hh inhibitor GDC-0449, we replaced its sulfonamide group with two methylpyridine-2yl at amide nitrogen to synthesize MDB5. We compared the Hh pathway inhibition and anti-fibrotic effect of MDB5 with GDC-0449 in vitro. Next, we developed MDB5 loaded micelles using our methoxy poly(ethylene glycol)-blockpoly(2-methyl-2-carboxyl-propylene carbonate-graft-dodecanol (PEG-PCC-g-DC) copolymer and characterized for physicochemical properties. We evaluated the therapeutic efficacy of MDB5 loaded micelles in common bile duct ligation (CBDL) induced liver fibrosis, mouse model. We also determined the intrahepatic distribution of fluorescently labeled micelles after MDB5 treatment.

**Results:** Our results show that MDB5 was more potent in inhibiting Hh pathway components and HSC proliferation in vitro. We successfully developed MDB5 loaded micelles with particle size of 40 ± 10 nm and drug loading up to 10% w/w. MDB5 loaded micelles at the dose of 10 mg/kg were well tolerated by mice, without visible sign of toxicity. The serum enzyme activities elevated by CBDL was significantly decreased by MDB5 loaded micelles compared to GDC-0449 loaded micelles. MDB5 loaded micelles further decreased collagen deposition, HSC activation, and Hh activity and its target genes in the liver. MDB5 loaded micelles also prevented liver sinusoidal endothelial capillarization (LSEC) and therefore restored perfusion between blood and liver cells.

**Conclusions:** Our study provides evidence that MDB5 was more potent in inhibiting Hh pathway in HSC-T6 cells and showed better hepatoprotection in CBDL mice compared to GDC-0449.

## Introduction

Liver injury stimulates hepatic stellate cells (HSCs) to transdifferentiate from a quiescent state to become proliferative, migratory and fibrogenic myofibroblasts (MF). In acute liver injury, MF-HSCs help to regenerate liver effectively, but persistent injury enhances their undesired accumulation and results in progressive fibrosis, defective repair and ultimately, cirrhosis 1. Several cytokines (e.g., IL-2, IL-6, and TNF-α), growth factors {e.g., transforming growth factor beta (TGF-β), fibroblast growth factor (FGF), and platelet-derived growth factor beta (PDGF-β)}, and metabolic pathways (e.g., kynurenine (Kyn), glutaminolysis, and TCA cycle) dictate HSC activation and proliferation 2.

Hh pathway plays an essential role in embryonic development and several pathological conditions such as congenital disabilities, cancers, and fibrosis 3. Hh signaling is not active in healthy adult livers, but it gets activated in response to a variety of liver insults and regulates tissue repair 4. Hepatocytes, HSCs, cholangiocytes, sinusoidal endothelial cells, and several immune cells are Hh ligand producer/effector 5. Recently, the role of Hh signaling in adipogenesis and metabolism has been established 6. During the repair mechanism, the Hh pathway promotes the Hippo pathway via activating Yes-associated protein 1 (YAP1) within HSCs 7. YAP1 activity in HSCs is glutaminolysis dependent, which is a process of converting glutamine to alpha-ketoglutarate (α-KG) by glutaminase (GLS), glutamate dehydrogenase (GDH), and transaminases. Glutaminolysis favors HSCs with high α-KG and enhances the TCA cycle by increasing ATP and key metabolite pools for the biosynthesis of nucleic acids, amino acids, and lipids to transdifferentiation into MF-HSCs. Blockade of Hh signaling in MF-HSCs has been shown to suppress its growth by regulating glutaminolysis through disrupting Hh-YAP1 signaling. Further, YAP1 also reprograms hepatocyte glutamine metabolism for liver regeneration and has been shown to promote hepatocellular carcinoma (HCC) cell growth and migration 8.

Liver sinusoidal endothelial cells (LSEC) are highly specialized endothelial cells lining the liver sinusoid with open fenestrae and absence of diaphragm and lack of basement membrane 9. Following liver fibrosis, LSEC capillarization leads to an increase in endothelial thickness (de-differentiation), basal lamina and collagen deposition, and decrease in sinusoidal porosity 10. Further, differentiated LSECs in paracrine fashion prevent activation of HSCs via nitric oxide (NO) in normal liver, but capillarized LSEC cannot prevent HSC activation. Increased Hh signaling in LSECs of injured liver promotes capillarization process, and its inhibition prevents LSEC capillarization and, thus improve exchange between blood and fibrotic liver 5.

Activation of the Hh pathway includes multiple steps providing an opportunity for regulating its activity through pharmacological intervention 11. Vismodegib (GDC-0449) and sonidegib (LDE225) are the two Food and Drug Administration (FDA) approved Smoothened (SMO) inhibitors for the treatment of advanced and metastatic basal cell carcinoma (BCC) 12. Previously we observed that although GDC-0449 can inhibit liver fibrosis progression in combination with other drugs such as peroxisome proliferator activator gamma agonist (PPAR-γ) rosiglitazone, and antifibrotic miR-29b1, alone its efficacy was modest in common bile duct ligation (CBDL) murine model 13. Therefore, we synthesized a series of GDC-0449 analogs for enhancing its therapeutic index and half-life with reduced toxicity. Based on the structure activity relationship (SAR), we substituted the sulfonamide with an aromatic or heterocyclic ring to provide additional hydrophobic interactions with the binding site of SMO. Specifically, the addition of a pyridine ring provided additional H-bonding site within the receptor binding cavity. Thus, we explored this position and substituted it with two methylpyridine-2yl at the same amide nitrogen, which gave the most potent compound of the series, 2-chloro-N^1^-[4-chloro-3-(2-pyridinyl)phenyl]-N^4^, N^4^-bis(2-pyridinylmethyl)-1,4-benzenedicarboxamide (abbreviated as MDB5). When mice bearing subcutaneous pancreatic cancer were treated with MDB5 loaded nanoparticles, there was a significant reduction in tumor growth, proliferation marker Ki-67, but increase in cleaved Caspase-3 and E-cadherin compared to GDC-0449 loaded nanoparticles treated group 14.

Herein, we evaluated the antifibrotic effect of MDB5 compared to GDC-0449 *in vitro* and *in vivo*. MDB5 showed better inhibition of Hh signal transduction and its direct GLI target genes. CBDL leads to the accumulation of bile acids within hepatocytes, which causes mitochondrial dysfunction, endoplasmic reticulum stress, and immune cell infiltration that can ultimately lead to inflammation, cell death, and liver injury 15. Systemic administration of MDB5 loaded micelles in CBDL mice decreased Hh pathway, sinusoidal capillarization, and collagen deposition in the liver compared to GDC-0449. We also observed significant downregulation of GLS1 by MDB5 loaded micelles compared to GDC-0449 loaded micelles, suggesting MDB5 is more efficient in inhibiting liver fibrosis and glutaminolysis.

## Methods

**Reagents.** GDC-0449 (Vismodegib) was purchased from LC Laboratories (Woburn, MA). TaqMan reverse transcription reagent kit was purchased from Life Technologies (Grand Island, NY). Radioimmunoprecipitation assay (RIPA) buffer and SYBR green-1 were purchased from Roche (Indianapolis, IN). 2, 2-Bis (hydroxymethyl) propionic acid, methoxy poly (ethylene glycol) (mPEG, Mn = 5,000, PDI = 1.03), stannous 2-ethyl hexanoate (Sn(Oct)_2_), and benzyl bromide), triethanolamine (TEA), hydroxybenzotriazole (HOBt), 1-[bis(dimethylamino)methylene]-1*H*-1,2,3-triazolo[4,5-*b*] pyridinium 3-oxid hexafluorophosphate) (HATU) and dodecanol were purchased from Sigma Aldrich (St. Louis, MO). VetScan Preventive Care Profile Plus was purchased from Abaxis (Union City, CA). Anti-glutaminase1 (anti-GLS1) (ab93434), anti-E-cadherin (ab231303), anti-CD31 (ab28364), and anti-Ki67 (ab15580) were purchased from Abcam, while anti-Gli1 (sc-149), anti-β-actin (sc-47778), and anti-YAP1 (sc-271134) were purchased from Santa Cruz Biotechnology, and anti-PTCH1 (AV44249), and anti-αSMA (ABT1487) from MiliporeSigma. All other reagents were obtained from Sigma Aldrich and used without further purification.

**Synthesis and characterization of MDB5**. All analogs of GDC-0449 were synthesized in two steps and characterized as described previously 14. In the first step to synthesise MDB5, to a mixture of 3-chloro-4-(methoxycarbonyl) benzoic acid (C_9_H_7_ClO_4_, 540 mg, 2.5 mmol), bis(2-pyridylmethyl)amine (C_12_H_13_N_3_, 740 mg, 3.7 mmol), and triethylamine (TEA, 505 mg, 5 mmol) in acetonitrile (25 mL) was added HATU (1.43 g, 3.75 mmol). The mixture was stirred at room temperature for 17 h and then evaporated to dryness. The residue was diluted with CH_2_Cl_2_ (50 mL) and extracted with brine (3 × 50 mL). The organic layer was dried over MgSO_4_, filtered, and concentrated. In the second step, a mixture of the crude amide and potassium hydroxide (KOH, 291 mg, 5.2 mmol) in CH_3_OH (25 mL) was heated at 50 °C for 17 h and cooled to room temperature. After removal of the solvent, the residue was purified by crystallization from acetonitrile (CAN, 50 mL) to give the desired potassium salt as a white solid. In step three, to a mixture of the potassium salt and 4-chloro-3-(pyridine-2-yl) benzenamine (197 mg, 0.96 mmol) in dimethylacetamide (DMA,5 mL) HATU (547 mg, 1.4 mmol) was added. The mixture was stirred at room temperature for 17 h and then quenched with brine (50 mL). The resulting precipitate was collected by filtration and dried. The crude product was purified by chromatography (silica gel, EtOAc/ACN = 50:50) to afford the desired diamide (149 mg, 10%) as a white solid. Mp 89-91 °C. The chemical structure was determined by ^1^H NMR (Bruker), the molecular weight was determined by Mass spectroscopy (Orbitrap Fusion™ Tribrid, Fisher Scientific), and purity was determined by HPLC analysis (Shimadzu Corp.).

**Cell culture**. In this study, we used immortalized hepatic rat stellate cell line (HSC-T6 cells). Cells were maintained in DMEM medium (Gibco, NY) supplemented with 10% fetal bovine serum (FBS) and penicillin-streptomycin (100 IU/ml and 100 mg/ml media, respectively at 37°C in a humidified environment of 5% CO_2_. For cell viability assay, 5 × 10^3^ HSC-T6 cells per well were seeded in 96-well plates and incubated overnight in 5% CO_2_ at 37 °C in a 95% humidified atmosphere. Cells were treated with concentration gradients of GDC-0449 analogs (MDB1-6, 0-200 µM in DMSO) and 0.25% of DMSO was used as a control. Cell viability was assessed by MTT (0.5 mg/mL) assay after 48h. For apoptosis assay, cells (3 × 10^5^/well) were seeded in a 6 wells plate overnight. The cells were then treated with 50 µM of GDC-0449 or MDB5 and incubated for 48h. Subsequently, both live and dead cells were collected, washed twice with cold PBS and the pellets were suspended in 500 µL binding buffer containing 5 µL of Annexin-V FITC and 1 µL of propidium iodide (PI) (100 µg/mL) (Alexa Fluor^®^ 488 Annexin V Apoptosis Kit, Invitrogen). The mixture was incubated for 15 min in the dark at room temperature and analyzed by flow cytometry. For cell cycle analysis, after harvesting total cells, the pellets were suspended in 1 mL of cold 70% ethanol and incubated overnight at 4 °C for fixation. Cells were washed twice with cold PBS and suspended in 500 µL FxCycle PI/RNase staining solution (Molecular Probes Inc., OR) for 30 min at RT in the dark and analyzed by a flow cytometer.

**Oxygen consumption rate and glucose uptake assay.** To assess stress induced by Hh inhibitors, mitochondrial oxygen consumption rates (OCR) were determined using OCR assay (Cayman Chemical, Ann Arbor, MI). Briefly, HSC-T6 cells were seeded in a black-walled 96 well plate (40,000 cells/well) and cultured at 37 °C with 5% CO_2_ for 12 h. Cells were treated with GDC-0449 or MDB5 (50 μM) or the vehicle (0.1% DMSO) in complete medium for 24 h. Subsequently, spent culture media was replaced with complete fresh media, and 10 µM phosphorescent oxygen probe solution was added. HS mineral oil (100 µL) was gently overlayed, and the plate was read at excitation 380 nm and emission 650 nm kinetically for 120 min. In separate wells (3 wells each without cells), 2 μM oligomycin, 2 μM phosphorescent oxygen probe, and 0.5 μM antimycin A was used as a control. For measuring the effect of MDB5 treatment on glucose uptake HSC-T6 cells were cultured and treated as above but in glucose free media (n=5). Before ending the experiment, cells were incubated with 2-[*N*-(7-nitrobenz-2-oxa-1,3-diazol-4-yl)amino]-2-deoxy-D-glucose (2NBDG) for 30 min and plates were centrifuged at 400×g for 5 min. Cells were washed with cell based assay buffer (Cayman) and gently added 100 µL of it, and the plate was read at excitation 485 nm and emission 535 nm. In separate wells (n=5) 200 µg/mL Apigenin treatment was used as a control.

**Transglutaminase activity assay.** Transglutaminase activity of HSC-T6 cells was determined after 24 h of treatment using a colorimetric assay kit (Biovision, #K571-100) as per manufacturer's instructions. For sample preparation, Dithiothreitol (DTT) 0.2 mM final concentration and protease inhibitor cocktail was added to the homogenization buffer. To prepare cell extract, 200 µL cold homogenization buffer was added to 2 × 10^6^ cells and disrupted by five cycles for freezing and thawing. For tissues, 100mg of frozen (stored at -80°C) was added 500 µL of cold homogenization buffer and processed in a bead mill. Cell/tissue homogenate was centrifuged at 16,000 × g in an Eppendorf tube for 20 min at 4°C. Lysate concentration was measured using BCA assay. Transglutaminase reaction mixture was prepared (50 µL/well) for each sample, and background control as directed (25 µL TG assay buffer, 10 µL donor substrate, 10 µL acceptor substrate, 1 µL of 1M DTT, 4 µl deionized water). Hydroxamate standards in a concentration range of 0 -10 mM were also prepared separately. The samples (50 µL) were mixed with of 50 µL reaction mixture and incubated at 37°C for 2 h. Next, 50 µL/well of the stop solution was added to all samples and background controls and centrifuge at ~1800 × g for 15 min. Absorbance (OD 525 nm) of the supernatants was measured using a plate reader. Sample's transglutaminase activity was measured by using the equation:


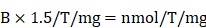


Where: B is hydroxamate amount from Standard Curve (nmol). 1.5 is nmoles of hydroxamate product generated in 150 µL reaction volume, T is the incubation time in minutes, and mg is the amount of protein/reaction in milligrams.

**Matrix assembly assay.** The matrix assembly capability of HSCs in the presence of MDB5 was determined. Briefly, cells were seeded in a clear bottom black 96-well plate (40,000/well) for 12 h. DMSO control, GDC-0449, and MDB5 were added to each well. Following 1 h incubation, Alexa Fluor 488 fluorescent labeled human plasma fibronectin (A488-FN) (11 μg/mL) was added, and incubated for 24 h. Cells were washed with Hank's Balanced Salt Solution (HBSS) containing Ca^2+^ and Mg^2+^ to remove non-assembled fibronectin (FN). HBSS solution (60 μL) was added to each well, and fluorescence signal was measured using a microplate reader (M5/M5e, SpectraMax) at excitation: 485 nm, and emission: 538 nm. To quantitate cell viability, a 30 μL aliquot of HBSS was removed from each well and was replaced with 30 μL of a luminescence reagent (CellTiter-Glo), and Luminescence was quantified immediately using the same plate reader. The average of fluorescence values of A488FN was subtracted from the control and drug-treated groups and was further normalized using each well's corresponding luminescence value. Lastly, the cell viability normalized fluorescence values were expressed as a percentage of the untreated control group 16.

**Preparation and characterization of micelles.** Drug-loaded micelles were prepared using methoxy poly(ethylene glycol)-blockpoly(2-methyl-2-carboxyl-propylene carbonate-graft-dodecanol (mPEG-b-PCC-g-DC) copolymer. It is an amphiphilic copolymer, which can self-assemble in micelles and encapsulate hydrophobic small molecules. The PEG part of this polymer forms corona of the micelles, and DC pepdent groups on the carbonate chain help in their self essambly. Micelles were prepared by film hydration as described 11 Briefly, 30 mg copolymer was dissolved in dichloromethane (DCM) containing 3.0 mg GDC-0449 or MDB5. The solution was then evaporated to make a thin film on glass vial and dried overnight under vacuum. PBS (pH 7.4) was added (4.0 mL) and probe sonicated at 30 W for 5 min on an ice bath. Micelles were concentrated using Amicon Ultra-15 Centrifugal Filter Unit (Millipore-Sigma) and sterilized using a syringe filter (0.2 µm).

The mean particle size and size distribution of the micelles were determined by dynamic light scattering using a Zeta Sizer^TM^ (Malvern Nano-ZS90, MA). Transmission electron microscopy (TEM) further confirmed micelle morphology and size. Increase in drug solubility was determined by HPLC (Shimadzu, Japan). Briefly, GDC-0449 and MDB5 loaded micelles with 0.5 mg theoretical drug encapsulation were dissolved in DCM for drug extraction using a bath sonicator for 30 min at RT. DCM was evaporated, and acetonitrile was added to dissolve residues and injected into HPLC equipped with reverse phase C-18 Inertsil ODS^®^ column (150 mm × 4.6 mm, 5 μm) (GL Sciences Inc.). Mobile phase composition was acetonitrile and water (60/40, v/v) at a flow rate of 1.0 mL/min. Detection wavelength of 230 nm and 266 nm were used for GDC-0449 and MDB5, respectively. For drug release study, micelles containing 0.5 mg of MDB5 were placed in a dialysis bag (1,000 Da) and placed in 50 mL of PBS (pH 7.4) containing 1.0% (w/w) Tween 80 (sink conditions). Samples were placed on an orbital shaker at 37°C, with constant shaking (100 rpm). Aliquots (1 mL) were taken at regular time intervals and replaced with fresh PBS (pH 7.4) containing 1.0% (w/w) Tween 80. The drug content in the samples was analyzed by HPLC as described above. Cell toxicity of micelles loaded with GDC-0449 and MDB5 was determined as described above.

**Animal experiments.** All animal experiments were performed according to the protocol approved by the Institutional Animal Care and Use Committee (IACUC) at the University of Nebraska Medical Center (UNMC), Omaha, NE. Adult (8-10 weeks old) C57BL/6 wild-type (WT) male mice were purchased from The Jackson Laboratory (Bar Harbor, ME). The mice were maintained on a 12h light-dark schedule and had free access to normal chow and water. Liver fibrosis was induced by CBDL as described previously 15. After 3 days of CBDL, mice were divided randomly into the following four groups (n=5/group): control (sham-operated), CBDL treated blank micelles, CBDL treated with GDC-0449 loaded micelles at a dose of 10 mg/kg, CBDL treated with MDB5 loaded micelles at a dose of 10 mg/kg. Micelles were administered 3 times a week for 2 weeks via intravenous tail vein injection. After treatment, animals were euthanized, and blood, as well as major organs, were collected. Serum was collected by centrifugation and used for biochemical analysis. Liver and other organs were either snap frozen (for immunohistofluorescence, Western blot, RT-PCR), or fixed in 10% neutral buffered formalin (for immunohistochemistry).

**Measurement of serum enzyme levels and liver histology.** Liver injury markers such as ALT, AST, TBIL levels in the serum of treatment groups were determined by using Preventative Care Profile Plus kit on a VetScan-2 (VS2) 17. To study the liver histology, specimens from different treatment groups were fixed in 10% paraformaldehyde buffered solution for 24h and embedded in paraffin using a standard protocol. Liver sections of ~5 mm thickness was prepared and stained with hematoxylin and eosin (H & E) for histological analysis, Masson's Trichrome and Sirius red for determining the levels of collagen deposition. Slides were scanned at 40X using iScan HT Slide Scanner (Ventana Medical Systems, Inc., AZ). The fibrotic area from five random views of Masson trichrome stained sections from each sample (n=5/group) was calculated with the Image J 1.44s software. The percentage of the fibrotic area was then calculated by comparing the collagen stained area to the total area.

**Hydroxyproline assay.** Hydroxyproline content of liver samples was determined using the hydroxyproline assay kit (# K226-100, Biovision, Mountain View, CA) according to the manufacturer's instructions. Briefly, liver tissues (50 mg) were homogenized using a bead mill homogenizer and then hydrolyzed with 10 N HCl. Next, 10N NaOH was added to neutralize, and the sample hydrolysate was dried and oxidized with an oxidation reaction mix. The bright-colored chromophore was developed with proprietary acidic developer and absorbance was measured at 560 nm. Hydroxyproline concentration was calculated from a standard curve prepared with pure hydroxyproline and expressed as mg hydroxyproline per gram of liver tissue.

**Immunohistochemistry of liver tissue.** Formalin-fixed liver sections of 5 µm thick were immunostained for α-SMA, GLS1, GLI1, PTCH1, and Ki-67, and E-cadherin as described earlier 18. Slides were scanned at 40 × (Ventana iScan HT), and the positive stained area was quantified by Image J software. For immunofluorescence staining, sections were incubated overnight with anti-αSMA (1:200), and anti-GLI1 (1:100), and anti-GLS1 (1:100) primary antibodies at 4°C. Slides were washed with tris-buffered saline containing (0.05% w/w) Tween 20 (TBST), and anti-rabbit or anti-goat secondary antibodies (IgG - DyLight^®^ 488 (Abcam) for 2 h at room temperature. To exclude the potential off-targeting bindings resulting from the secondary antibodies, a negative control was stained in parallel without the use of primary antibodies. Sections were visualized under a fluorescent microscope; staining was quantified by Image-J software using at least 4 randomly chosen fields at ×4 magnification per section for each mouse.

**Scanning electron microscopy (SEM) of liver tissue.** SEM of liver tissue was carried out as previously described with some modification 5. Briefly, after treatment with micelles mouse livers were perfused through the portal vein with a fixative containing 1.5% glutaraldehyde (Electron Microscopy Sciences) in 0.1 M cacodylate buffer, pH 7.4. Liver samples were cut into small cubes and fixed in formaldehyde for an hour, then washed in Sorenson's phosphate buffer, and post-fixed in 1% osmium tetroxide in water. They were then washed in buffer, dehydrated in a graded ethanol series, and subsequently air-dried using hexamethyldisilazane (Electron Microscopy Sciences). Dried tissue was mounted on aluminum stubs with double-sided carbon tape. A thin layer of gold-palladium was applied with a Hummer VI Sputter Coater (Anatech USA). Samples were imaged at 25kV in an FEI Quanta 200 SEM operating in high vacuum mode. For quantification of total sinusoidal area, images of 10 lobular sinusoids per mouse were measured for porosity using MetaMorph software (version 7.0; Molecular Devices, PA). Within a defined region of representative sinusoids, total sinusoidal area and the open area of individual fenestrae were quantified in square microns (n= 20).

**Intrahepatic distribution of micelles.** For biodistribution studies, Cy7.5 fluorescent labeled scrambled oligonucleotide (Oligo) (2mg/kg) was co-formulated in micelles with GDC-0449 or MDB5 (10mg/kg) using our methoxy poly(ethylene glycol)-blockpoly(2-methyl-2-carboxyl-propylene carbonate-graft-dodecanol-graft-tetraethylene-pentamine (mPEG-PCC-g-DC-g-TEPA) copolymer 15. Five animals per group were tested. In separate groups after completion of 5 injections of therapeutic study as above, micelles loaded with MDB5 + Cy7.5 SCR-Oligo or GDC-0449 + Cy7.5 SCR-Oligo were injected i.v. After 4 h post-injection, mice were sacrificed, and livers were collected and stored at -80 °C until extraction and further analysis. Oligo distribution in liver was visualized under a confocal fluorescence microscope, while concentration in tissue sample homogenates was determined by quantification of fluorescence intensity (Ex: 670 nm, Em: 700 nm) 18.

**Quantitative real-time RT-PCR and Western blot analysis.** For real-time RT-PCR, RNA was extracted from the liver tissues using the RNeasy Mini Kit (Qiagen, MD) and reverse transcribed to cDNA using TaqMan RT kit (Carlsbad, CA). Gene expression levels were determined by two-step RT-PCR with a Light Cycler 480 (Roche, IA) using QuantiTect primer assays (Qiagen, MD) for different genes. All the real-time RT-PCR results were analyzed using comparative C_T_ method, and gene expression were normalized with β-actin gene as a control. To determine protein expression levels, snap frozen liver tissues were homogenized in RIPA buffer containing protease inhibitor cocktail. Equal amounts of protein were separated by SDS-PAGE gel electrophoresis on 4-15% Mini-PROTEAN polyacrylamide gel (Bio-Rad, CA) and transferred to PVDF membrane. After blocking with blocking buffer, the membrane was probed using primary antibodies against GLI1 (1:1000), PTCH1 (1:1000), α-SMA (1:1000), GLS1 (1:2000), and YAP1 (1:1000) overnight at 4°C. After washing, blots were probed with IR dye-conjugated corresponding secondary antibodies (LI-COR Biosciences, NE). The housekeeping gene β-actin (1:1000) was used as an internal loading control for normalization. Quantification was performed by measuring the intensity of the signals using Image-J software (version 3.0; NIH).

**Statistical analysis.** Data were presented as the mean ± S.D. The results were analyzed, and individual group means were compared using Student's unpaired t-test. A p-value of at least 0.05 was considered statistically significant.

## Results

### MDB5 Synthesis and its effects on HSC-T6 cells

**Figure 1:**
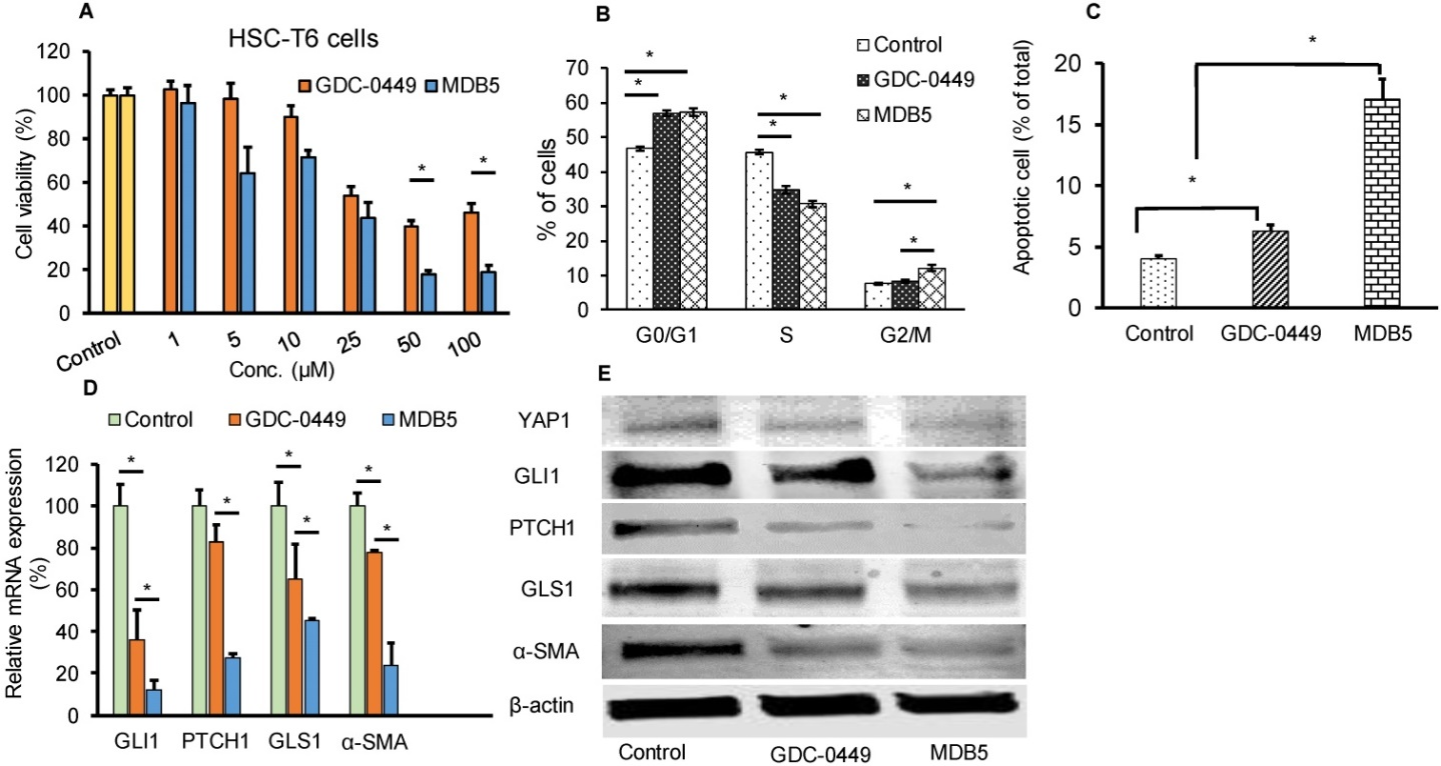
** Effect of MDB5 on cell viability, and cell cycle distribution of HSC-T6 cells. (A)** Cell viability was determined by MTT assay. The IC50 value of MDB5 was 25 µM compared to 50 µM of GDC-0449 in HSCs treated for 48 h. **(B)** Hh pathway inhibition altered the cell cycle profile in HSC-T6 cells. Cell apoptosis assay of each group using Annexin-V binding assays. Both the early-stage apoptotic cells (Hoechst 33342-positive and PI-negative cells) and late-stage apoptotic cells (Hoechst 33342-positive and PI-positive cells) were analyzed in our study. Similar results were obtained in three independent experiments and results were expressed as the mean ± SEM. A t-test was used to compare different groups, and p<0.05 was considered statistically significant. **(C)** % of apoptotic cells (*: p<0.05 between the two groups). **(D)** Gene expression assay with qPCR of Hh pathway regulated gene GLI1, PTCH1, GLS1, and α-SMA. A t-test was used to compare different groups, and p<0.05 was considered statistically significant. *: P<0.05 between the two groups. **(E)** Representative Western blot analysis.

We synthesized GDC-0449 analogs including MDB5 as described 14. MDB5 was characterized using ^1^H NMR, and HPLC analysis (Fig. S1). The cytotoxic effect of GDC-0449 analogs against HSC-T6 cells was estimated using MTT assay. Cells were treated with various concentrations of GDC-0449 and its analogs ranging from 1-200 µM in DMSO. As shown in Figure S2, MDB5 was the most potent among all the analogs, and treatment with MDB5 caused a significant decrease in the cell viability at 48 h at 50 and 100 µM compared to GDC-0449 (Fig. 1A). The 50% inhibitory concentration (IC_50_) value of MDB5 was 25 µM, while that of GDC-0449 was 50 µM at 48 h post incubation of HSC-T6 cells. The high inhibitory concentrations are in line with the recent report where IC_50_ of GDC-0449 was reported to be 138.46 µM in primary HSCs 19. Other than MDB5, none of the analogs had any noticeable cytotoxic effects on HSC-T6 cells at 48 h post incubation. Further, treatment with GDC-0449 and MDB5 clearly showed a change in the appearance of HSC-T6 cells compared to control cells. Control cells exhibited an elongated, flattened fibroblast-like shape (mesenchymal phenotype) (Fig. S3A), whereas drug-treated cells exhibited a more rounded star-like configuration with thin, slender, and shrunk abnormal morphology suggestive of reversal to a more quiescent state (decreased mesenchymal phenotype). Further, MDB5 treated cells also reached a statistically significant (P < 0.05) lower cell density after 24 h compared to control and GDC-0449 treated cells. We analyzed the effect of GDC-0449 and MDB5 on cell cycle progression by FACS analysis (Fig. S3B). The result showed that treatment with Hh inhibitors led to a significant increase in percentage of cells in the G_0_/G_1_ phase (46.7 ± 0.6% Vs. 56.89 ± 1.10% and 57.25 ± 1.23%) and G_2_/M phase (7.86 ± 0.3% Vs. 8.39 ± 0.3% and 12.9 ± 1.3%), compared to control, suggesting that both GDC-0449 and MDB5 exert their anti-proliferative effects by disturbing cell cycle. S phase was significantly reduced from 45.62 ± 0.7 in control cells to 34.78 ± 0.7% in GDC-0449 and 30.65 ± 0.1 % in MDB5 treatment (Fig. 1B).

Next, we examined whether GDC-0449 and MDB5 induced apoptosis are attributed to HSC-T6 cell death using Annexin V-Cy5/PI staining assay and the apoptotic cells were quantified by flow cytometry (Fig. S4). The percentages of early apoptotic, late apoptotic, necrotic, and live cells are shown in Figure 1C. The ratio of Annexin V and PI-positive cells treated with MDB5 was significantly higher (16.3%), compared to GDC-0449 treated cells (6.9%). Therefore, the results from the cell cycle and apoptosis assay studies were consistent with the MTT result.

### Effect of MDB5 on Hh pathway components

We assessed the Hh pathway and related components in HSCs. Shh after binding to the PTCH1 receptor, disrupts SMO inhibition and increases nuclear translocation of the transcription factor GLI1/2. Upon Hh inhibition with GDC-0449 (48 h), we observed a significant decrease in GLI1 and PTCH1 expression. Further, GLI1 and PTCH1 transcripts were significantly downregulated upon MDB5 treatment, relative to GDC-0449. Similarly, GLS1 and α-SMA transcripts were significantly downregulated upon MDB5 treatment, relative to GDC-0449 (Fig. 1D). WB results were also consistent with RT-PCR results, and we observed the same trend of decreased all these proteins after 48 h of treatment (Fig. 1E, Fig. S5).

### Effect of MDB5 on OCR, glucose uptake, fibronectin assembly, and transglutaminase activity

**Figure 2:**
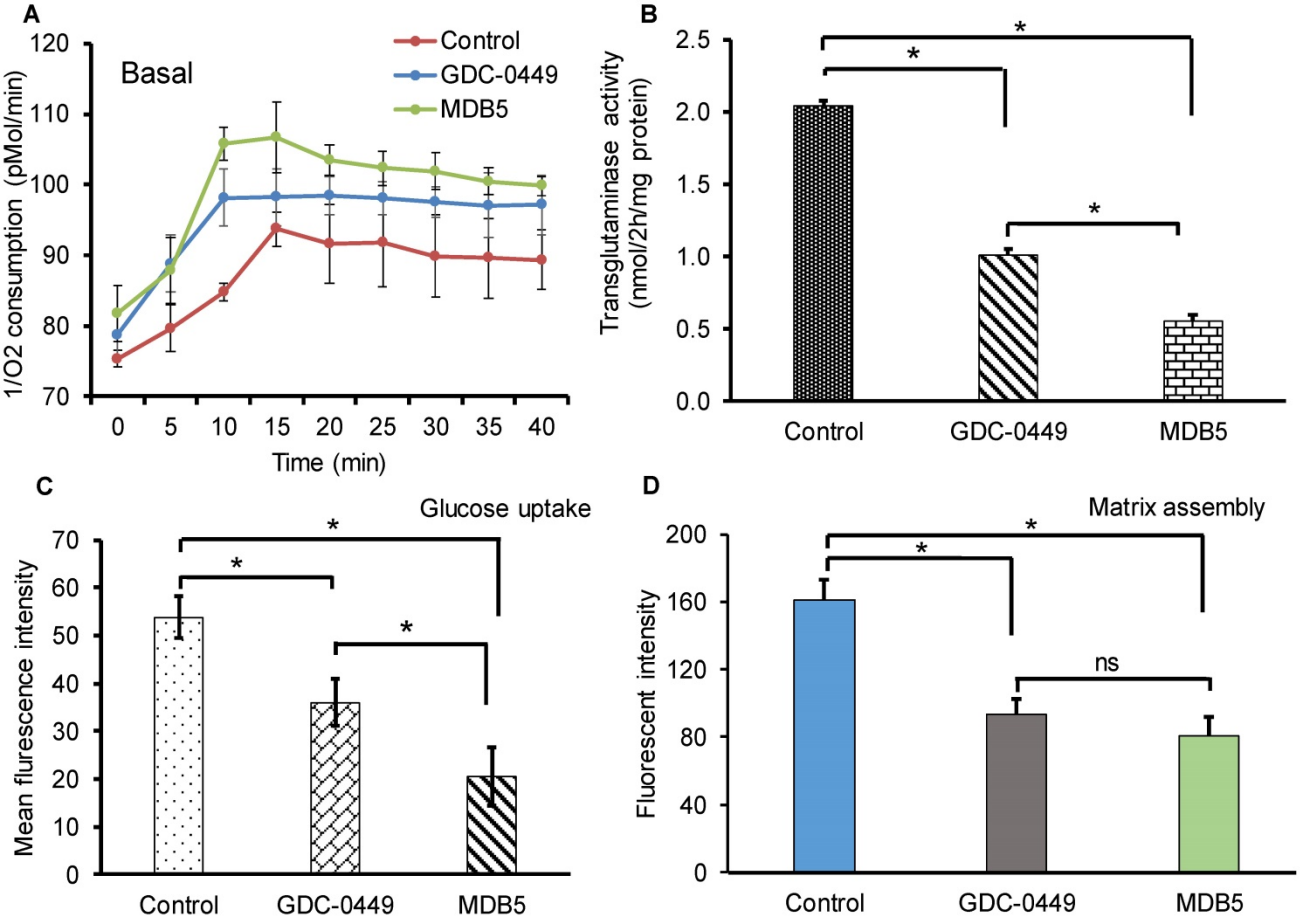
** Effect of Hh inhibitors on cellular metabolism, transglutaminase activity, and fibronectin matrix assembly. (A)** HSCs were treated with MDB5 and GDC-0449, and basal oxygen consumption rate (OCR) was determined after 24 h. **(B)** Transglutaminase activity was determined by colorimetric assay after treating cells with GDC-0449 or MDB5 at their IC_50_. **(C)** Glucose uptake study was determined using 2-NBDG kit after 24h treatment with GDC-0449 or MDB5. We found a correlation between the amount of transglutaminase activity and the extent of Hh pathway activation. **(D)** Fibronectin assembly assay of HSC-T6 cells treated with DMSO, GDC-0449 or MDB5 (50 µM) for 24 h. A t-test was used to compare different groups, and p<0.05 was considered statistically significant. *: P<0.05 between the two groups (n=5).

Increased metabolic activities of HSCs have recently been reported in liver fibrosis 20. Increased mitochondrial respiration results in a striking elevation in the oxygen consumption rate (OCR) of activated HSCs compared to the normal counterparts. We investigated the effect of Hh pathway inhibition on OCR by using phosphorescent oxygen probe-based assay. The probe signal is quenched by oxygen, resulting in a signal that is inversely proportional to oxygen present in a closed well. As shown in Figure 2A, the untreated HSC-T6 cells have markedly high O_2_ consumption over time compared to treated cells (less remainent O_2_ in air space over the cell). Treatment with GDC-0449 and MDB5 resulted in low oxygen consumption by cells, leading to high O_2_ present in air space over the cell. Because we observed that the Hh inhibition suppressed GLS1 expression in HSCs, we wanted to check GLS1 inhibition also results in decreased transglutaminase activity in these cells. As shown in Figure 2B, the glutaminase activity was indeed decreased after Hh inhibition. This was expected because the total enzymatic activity is dependent on the amount of GLS1 present, and Hh inhibition resulted in low GLS1 expression. We further confirmed that glucose uptake was significantly reduced after MDB5 treatment in HSC-T6 cells (Fig. 2C).

Matrix assembly is a process of self-association of ECM proteins to form fibrillar networks 21. At the injury site, when ECM glycoprotein fibronectin (FN) bind to cell surface receptor α5β1 integrin, its self-association is initiated by the N-terminal assembly domain. Bound FN undergo several conformational changes which expose additional binding sites and start the formation of insoluble thick fibril bundles. These fibrils form a closure to cover the bare wound surface, which helps epithelial cells migrate across the surface to repair the gap 22. HSCs produce most of the major and minor matrix to proteins of the fibrotic liver including fibronectin 23. Therefore, we determined the effect of MDB5 on FN assembly in the presence of HSC-T6 cells. The quantification of fluorescent intensity revealed that control cells assembled significantly more 488-FN-containing fibrillary matrix than GDC-0449 and MDB5 treated cells after 24 h of incubation with 488-FN (Fig. 2D). Together, these data reveal that in the presence of active Hh signaling in myofibroblasts assemble a mature fibrillar FN matrix more rapidly than in the presence of Hh inhibitors.

### Micelle formulation and characterization

**Figure 3:**
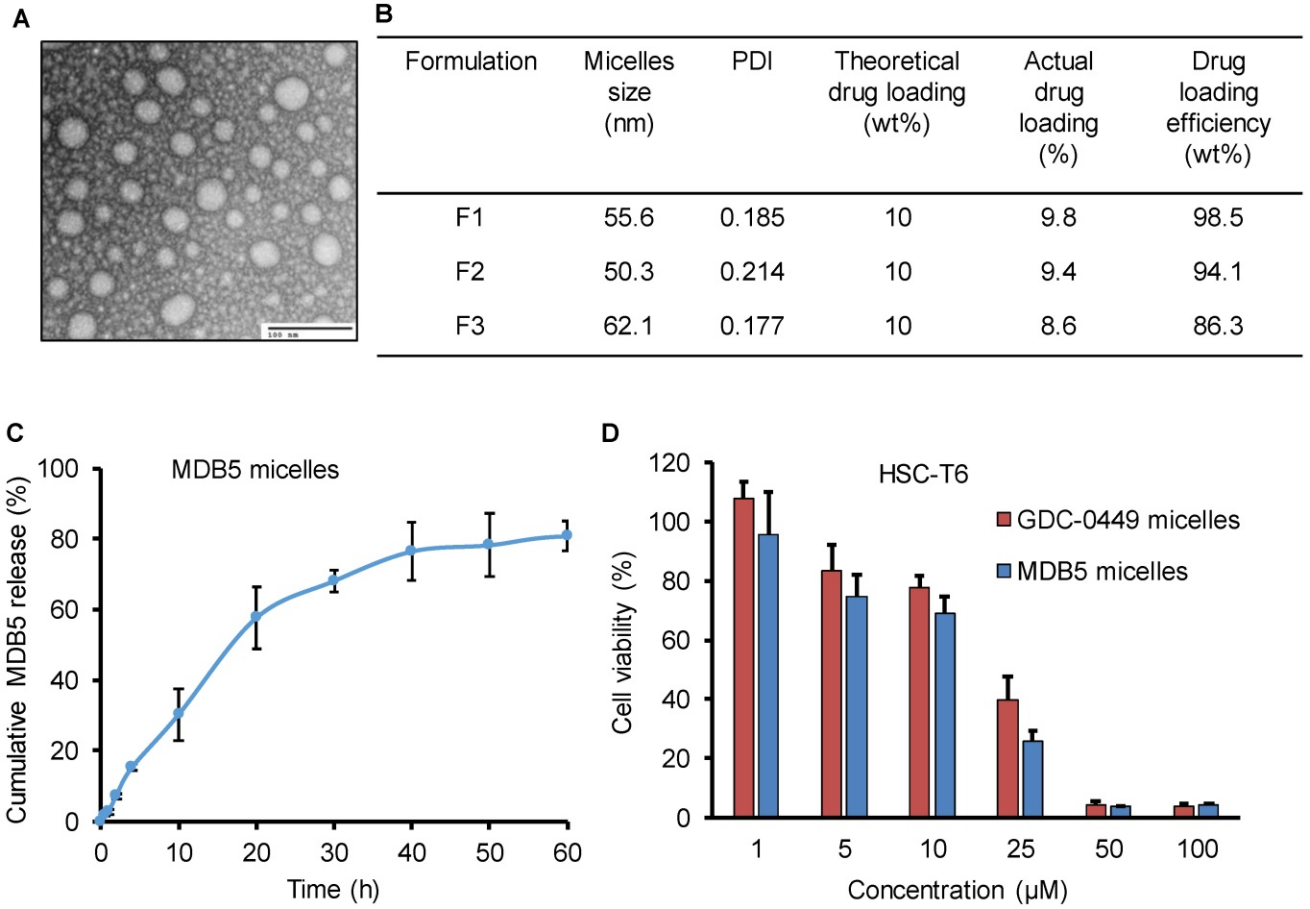
** Characterization of MDB5 loaded PEG-b-PCC-g-DC micelles. (A)** TEM image of MDB5 loaded micelles (scale bar = 100 nm). **(B)** Table representing the size and drug loading characterization of three independent formulations. **(C)** Cumulative MDB5 release from micelles in the medium (PBS + 1% w/w Tween 80) at pH 7.4 as sink conditions over a time period of 60 h (n=3). **(D)** Cell viability % determined at 48 h after drug loaded micelles exposure in HSCs (n=5).

PEG-PCC-DC copolymer was used to formulate the micelles for MDB5 and GDC-0449 by film hydration as described before 15. The micelles particle size and size distribution indicated as the hydrodynamic diameter was 50 ± 10 nm with a narrow size distribution as measured by dynamic light scattering. Transmission electron microscopy (TEM) picture of the micelles demonstrates that the particles were well-dispersed spheres with sizes ranging from 40 ± 10 nm diameter (Fig. 3A). Being a hydrophobic molecule, MDB5 was successfully loaded into micelles (up to 10% w/w) and micelles increased its aqueous solubility up to ∼2000 ± 50 μg (Fig. 3B). The *in vitro* release profile of the loaded MDB5 from the micelles at physiological pH is illustrated in Figure 3C. MDB5 released in a sustained manner, and around 60% of the total drug was released from the micelles at 24 h. GDC-0449 loading and release studies have been reported earlier 11. We determined the anti-proliferative properties of drug-loaded micelles in HSCs. Cell viability assays demonstrated that MDB5 and GDC-0449, when loaded in micelles, had higher efficacy (Fig. 3D), possibly by increased drug solubility of both drugs by micelles and enhanced micelles-mediated cellular uptake 19.

### Measurement of serum enzyme levels and liver histology

**Figure 4:**
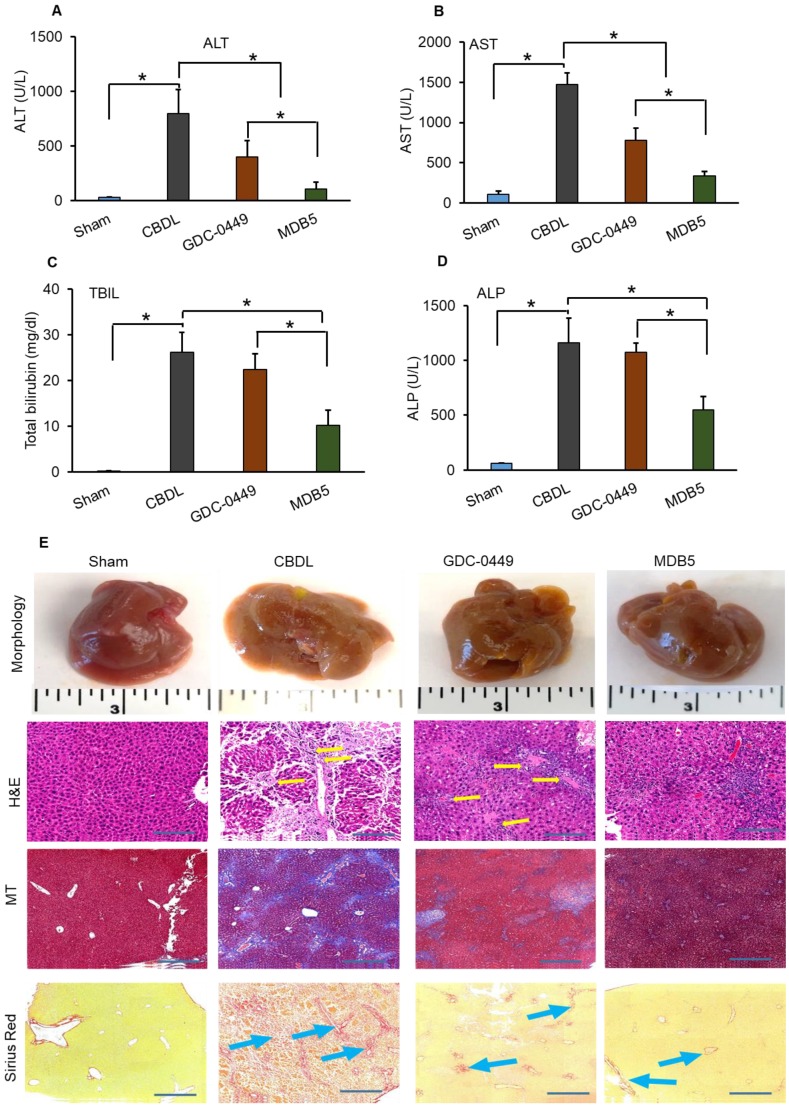
Effect of GDC-0449 and MDB5 loaded micelles in CBDL induced liver fibrosis.** (A)** Alanine transaminase (ALT).** (B)** Aspartate transaminase (AST). **(C)** Total bilirubin (TBIL).** (D)** Alkaline phosphatase (ALP) levels after treatment. Results are presented as the mean ± S.D. (*n* = 4). A t-test was used to compare different groups, and p<0.05 was considered statistically significant. *: P<0.05 between the two groups. **(E)** Representative macroscopic pictures of livers from CBDL mice after systemic administration of micelles loaded with GDC-0449 or MDB5 (upper first panel). H&E staining representing damaged liver architecture after CBDL (upper second panel, yellow arrows). Collagen specific Masson's trichrome (MT) (Third panels), and Sirius red staining (fourth panel) of liver sections. Treatment with GDC-0449 and MDB5 loaded micelles reduced collagen staining (original magnification, ×10).

Previously we evaluated the effects of GDC-0449 loaded micelles on hepatic histological damage. Micelles of both the drugs were well tolerated by mice, without visible sign of toxicity. Even after multiple dosing, no remarkable changes in general activity and body weight were observed, showing that micelles are well tolerated *in vivo*. Serum levels of ALT, AST, ALP, and TBIL were quantified with Preventative Care Profile Plus kit. The results showed that serum enzyme activities were elevated in CBDL mice compared with sham-operated (control) mice. The activity of liver enzymes in serum was decreased in mice treated with GDC-0449 loaded micelles. Further, this decreasing pattern was significantly enhanced in mice receiving MDB5 loaded micelles (Fig. 4A-D). Altogether, these data demonstrated that Hh inhibition protected mice from bile-induced liver injury, and MDB5 has a better antifibrotic potential compared to GDC-0449. Macroscopic examination of livers revealed that control animal livers had normal gross morphology, while CBDL mouse livers were grossly enlarged and cholestatic with a pitted surface. Treatment with GDC-0449 and MDB5 resulted in near normal liver morphology (Fig. 4E, upper first panel). Microscopic examination of H&E staining showed that CBDL caused noticeable hepatic pathological changes, including swollen and disorganized hepatocytes, loss of hepatic lobule structure, and sever vacuolation (Fig. 4E, second panel). GDC-0449 treatment could not fully reverse the damage. In contrast, the liver of MDB5 treated groups showed normal structure, and the hepatic cells were orderly arranged and comparable to the sham liver. Thus, MDB5 treatment significantly ameliorated histological damage by decreasing steatosis and hepatic lesions in CBDL mice compared to GDC-0449. Masson's trichrome and Sirius red staining showed that CBDL surgery led to the extensive accumulation of collagen in the liver tissues. Although GDC-0449 micelles treatment significantly decreased collagen deposition compared to CBDL, still fibrous bands were evident. The liver tissues of the sham and MDB5 loaded micelle treated groups showed only traces of collagen deposition in the walls of major blood vessels (Fig. 4E, third & fourth panel). Therefore, the protective effect of MDB5 micelles on liver injury and collagen accumulation was significantly higher than GDC-0449.

**Figure 5:**
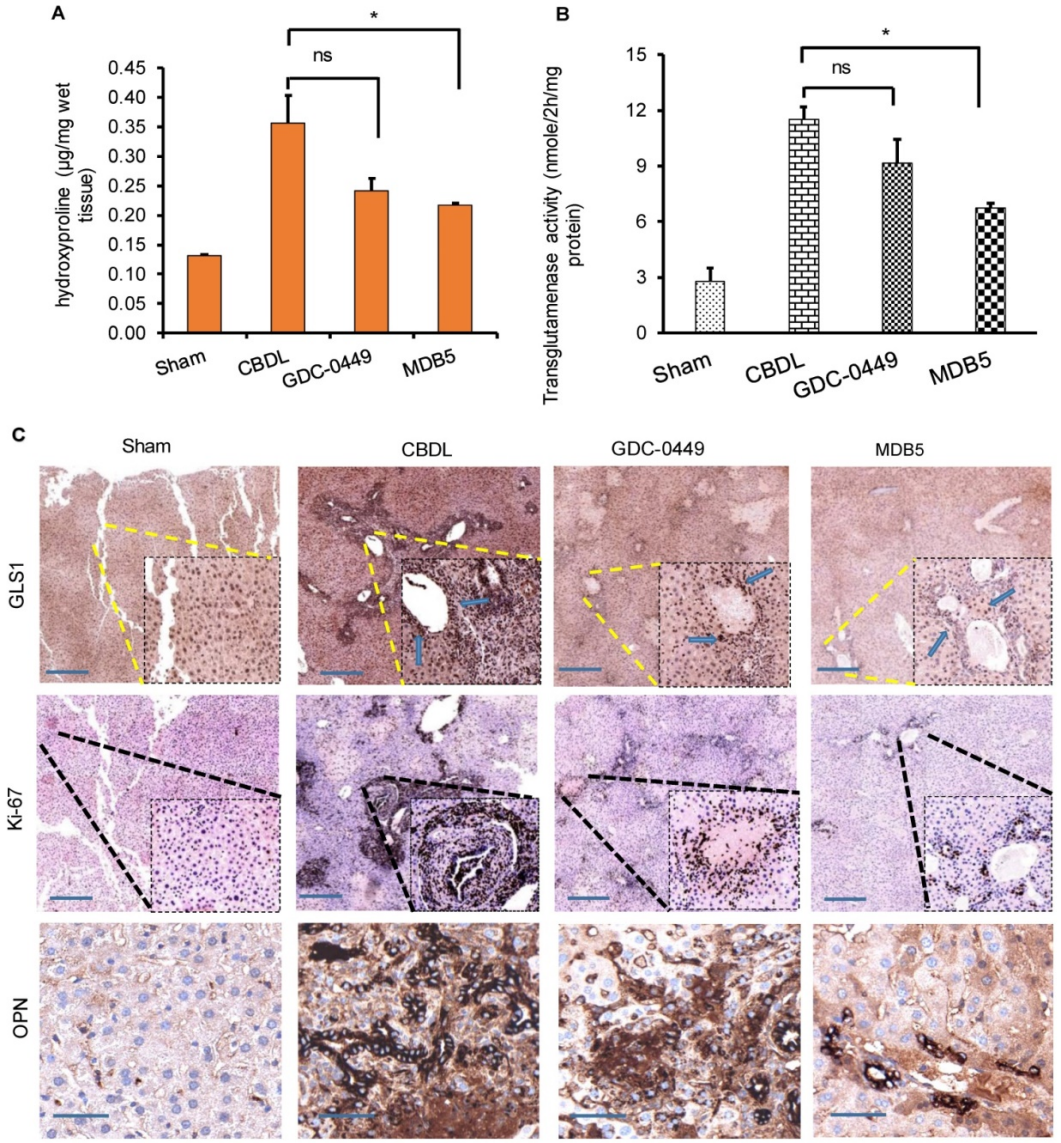
GDC-0449 and MDB5 loaded micelles inhibit progression of CBDL-induced liver fibrosis. **(A)** Hydroxyproline content.** (B)** Transglutaminase activity.** (C)** IHC staining for protein expression of GLS1 and Ki67 (upper and middle panels) (Objective 10X, inset objective 40 X) OPN (lower panel) in liver tissues (Objective 40X).

Hydroxyproline is a non-proteinogenic amino acid formed by post-translational hydroxylation of proline by prolyl hydroxylase. Typically, collagen fibers contains about 1/3rd of Gly and 1/4th of proline or hydroxyproline. The hydroxyproline content increases with increasing histological score in liver fibrotic patients. Higher hydroxyproline in collagen provides conformational rigidity and stabilize it by forming a hydrogen bond with main chain carbonyl groups. Therefore, we calculated hydroxyproline content among the different treatment groups. A significant increase in hydroxyproline content was evident liver tissue of CBDL animals (P < 0.05, Fig. 5A). As we previously reported, Hh inhibition reduces the level of collagen deposition in CBDL mice, here also we found that MDB5 loaded micelles significantly reduced collagen deposition in mice repeated statement.

In the epithelial cells, glutamate is converted into α-KG by GDH or transaminases, such as glutamate oxaloacetate transaminases (aspartate aminotransferase) and glutamate pyruvate transaminases 24. Hh signaling induces glutaminolysis for the increased energy demands of activated HSCs 25. We measured the liver tissue levels of transglutaminase after different treatments. As we can see in Figure 5B, transglutaminase activity was increased in the fibrotic liver probably due to increased GLS1 expression levels. Therefore, to confirm this, we determined GLS1 expression in CBDL mice compared to sham animals at protein levels. It is noteworthy that treatment with MDB5 significantly decreased GLS1 expression as well as its activity compared to CBDL, but not with GDC-0449 (Fig. 5C, upper panel). These results recapitulate our *in vitro* results discussed earlier. Upon CBDL, the proliferation of cholangiocytes trigger bile duct hyperplasia and increase the number of Ki-67^+^ cholangiocytes 26. When mouse liver tissues were stained with Ki-67, we observed a significantly higher number of Ki-67^+^ cells in CBDL group (Fig. 5C, middle panel). Thus, CBDL increased the proliferative activity in Hh-responsive hepatocytes and ductular cells. The overgrowth of cholangiocytes was significantly inhibited by the treatment with MDB5 exerting the most significant effect. OPN is a profibrogenic factor, which is regulated by GLI protein in the liver fibrosis 27. Therefore, we compared OPN expression in CBDL mice after various treatments. In untreated mice, CBDL caused a significant induction of OPN staining than sham mice. GDC-0449 loaded micelles treated mice exhibited decreased OPN expression than CBDL untreated mice. However, MDB5 loaded micelles treated mice showed even lower OPN expression than GDC-0449 (Fig. 5C, lower panel). Taken together, these data suggest that micellar delivery of MDB5 significantly attenuates liver fibrosis in mice.

### Effect of treatment on Hh activity and its target genes in the liver

**Figure 6:**
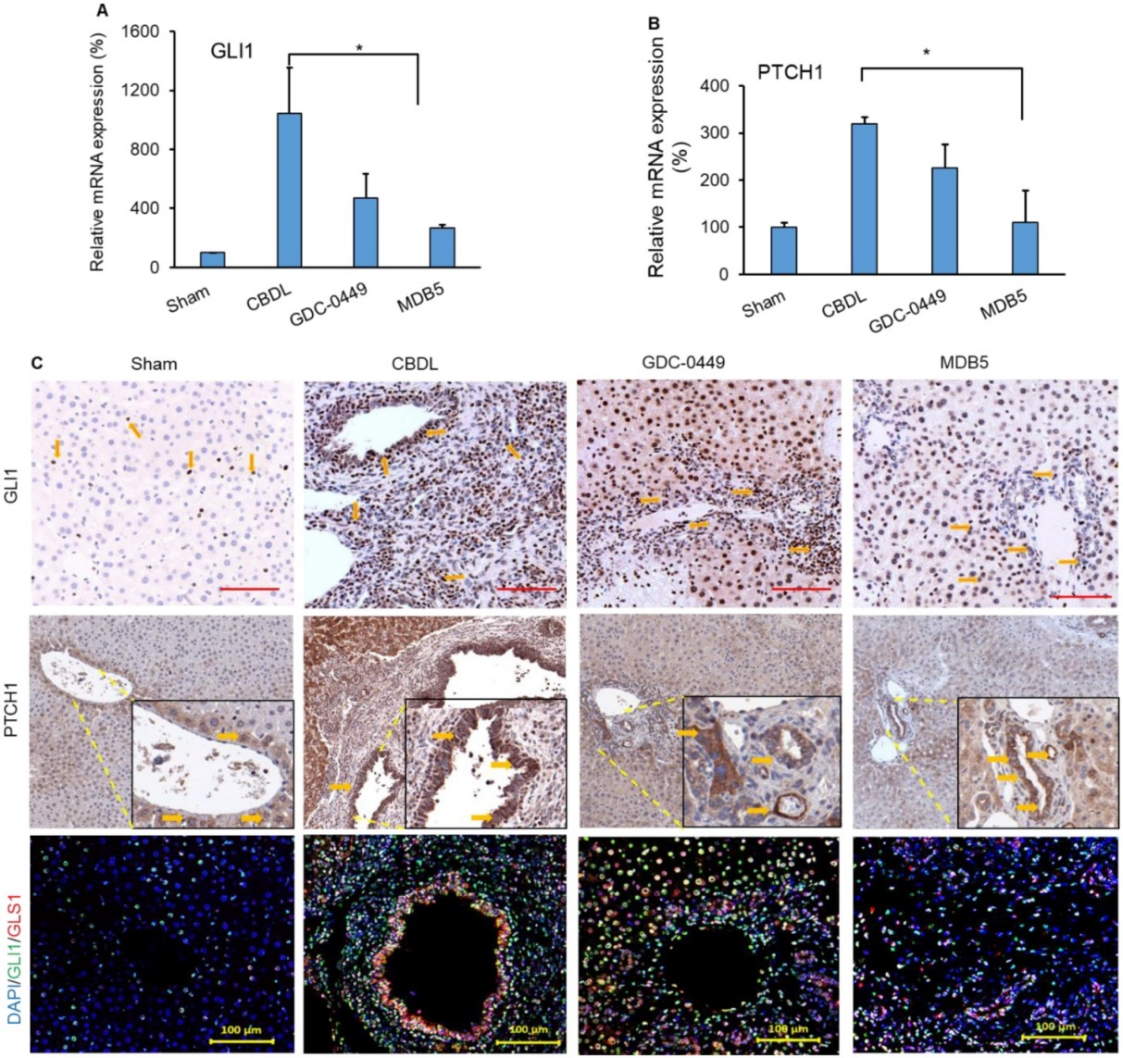
Hedgehog (Hh) pathway is activated during CBDL induced liver fibrosis. Relative liver mRNA levels of **(A)** GLI1, and **(B)** PTCH1 after treatment with GDC-0449 or MDB5 loaded micelles in CBDL mice. **(C)** Immunohistochemical (IHC) analysis for protein expression of GLI1 (Objective 20X) and PTCH1 (upper and middle panels) in mouse liver (Objective 10X, inset objective 40X). GLI1 (green) and GLS1 (red) were co-stained with IF (scale bar 100 µm).

Next, we determined the Hh components GLI1 and PTCH1 in mouse liver after treatment. CBDL mice had higher mRNA expression of Hh effector genes (GLI1, *P* < .0001 and PTCH1, *P* = .0214), than sham mice (Fig. 6A &B). Further, we also observed increased immunostaining for both genes (GLI1, and PTCH1) in CBDL mice (Fig. 6C, upper and middle panel). Their expression was significantly lowered upon MDB5 loaded micelles treatment as reflected by both RT-PCR, and immunostaining staining. Further, co-staining of GLI1 and GLS1 were found colocalized in these areas, indicating that HSCs up-regulate GLS1 upon Hh activation (Fig. 6C, lower panel). Consistent with previous results, both GLI1 and GLS1 expressions were decreased after treatment with Hh inhibitor loaded micelles in fibrotic liver.

### MDB5 treatment prevented EMT

**Figure 7:**
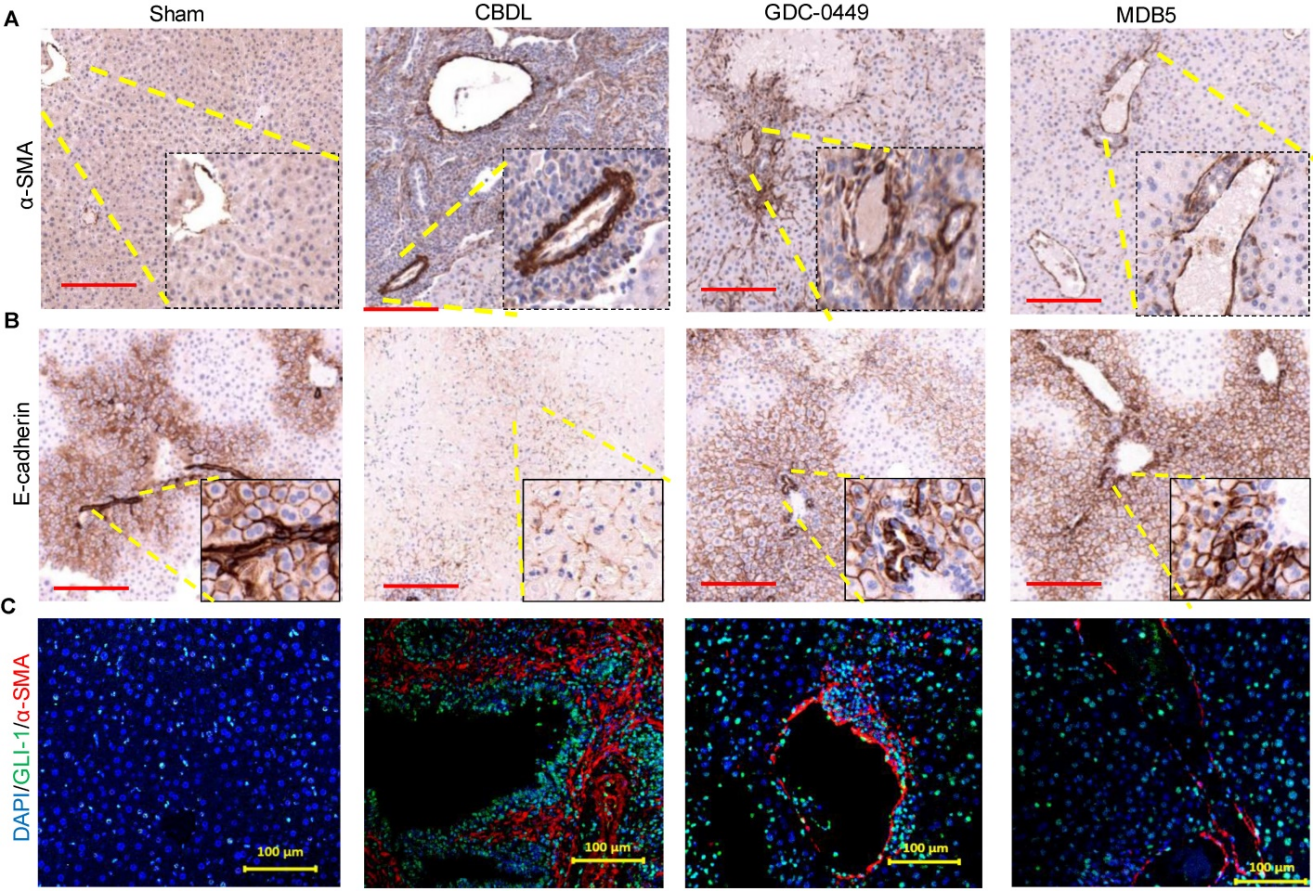
Effects of Hh signaling inhibition by MDB5 and GDC-0449 loaded micelles on protein expression of epithelial and mesenchymal markers in CBDL mice.** (A)** Representative IHC staining for determining levels of α-SMA Objective 10X, inset objective 40X, Scale bar 200µM). **(B)** E-cadherin (Objective 10X, inset objective 40X, Scale bar 200µM). **(C)** GLI1 (green) and α-SMA (red) were co-stained with IF (scale bar 100 µm).

The epithelial to mesenchymal transition (EMT) is an adaptive response of epithelial cells following chronic liver injury 13. During the EMT process hepatocytes and biliary epithelial cells lose their epithelial phenotypes (E-cadherin) and acquire mesenchymal characteristics (α-SMA) 28. Staining for α-SMA was increased in CBDL mice compared to sham mice (Fig. 7A). On the other hand, epithelial marker E-cadherin expression was lost upon CBDL mediated fibrosis induction (Fig. 7B). Treatment with micelles decreased α-SMA expression and restored E-cadherin suggesting these drugs have prevented EMT. GLI1 and α-SMA expression was perivascular co-localized in CBDL mice (Fig. 7C). The Gli1^+^ cells after injury differentiate into myofibroblasts in organ fibrosis and acquire expression of α-SMA 29. Thus, in our results we observed Gli1^+^ cells proliferate and differentiate into myofibroblasts in liver fibrosis. Treatment of CBDL mice with MDB5 led to a significant reduction in α-SMA, and GLI1 staining, corroborating the findings of a recent study 30.

### MDB5 treatment prevented LSEC capillarization

**Figure 8:**
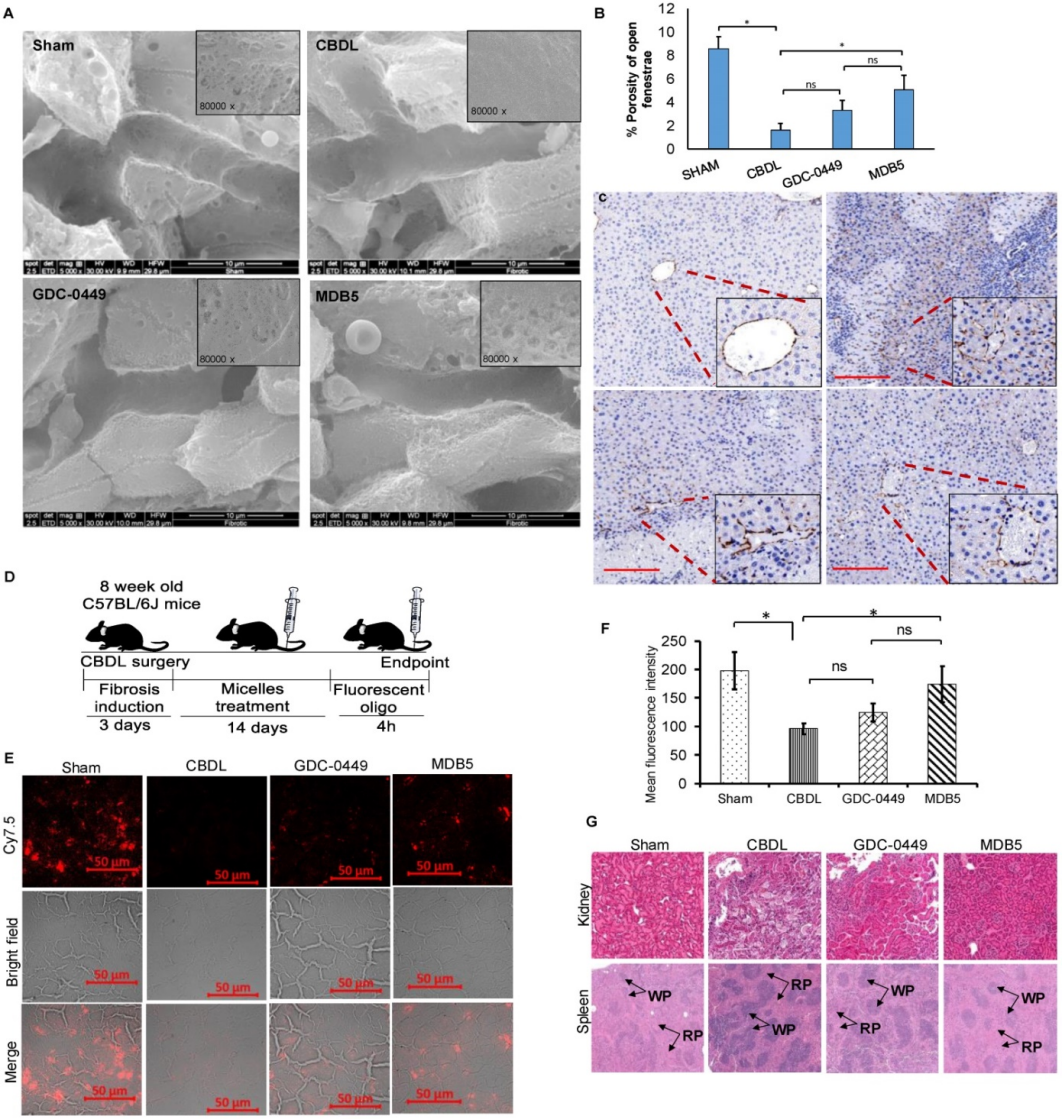
Effect of Hh pathway inhibition on LSEC capillarization and distribution of fluorescent micelles in the liver. **a** SEM of liver tissues (scale bar 10 µm, inset magnification= 80000X). **b** Percentage porosity of open fenestrae in different groups. **c** IHC staining for CD31 protein; a marker for capillarization (Objective 10X, inset objective 40X, scale bar 200 µM). **d** Experimental plan for biodistribution study. Micelles loaded with Cy5.5 labelled scrambled oligonucleotide were injected, and the liver tissues were collected 4 h post injection. **e** Representative image of liver slides showing fluorescent micelles for distribution. **f** Quantitation of fluorescent signal after extraction of oligonucleotide from different treatment groups. **g** H & E staining of kidney and spleen after two weeks of treatment with GDC-0449 or MDB5 loaded micelles. A t-test was used to compare different groups, and p<0.05 was considered statistically significant. *: P<0.05 between the two groups (n=5).

Capillarization is a process of losing LSEC fenestrae and formation of an organized basement membrane that precedes fibrosis. SEM showed that sham livers had sinusoid with open fenestrae, but fibrosis induction significantly defenestrated and formed continuous endothelium indicated capillarization (Fig. 8A). Inhibition of Hh pathway using GDC-0449 and MDB5 loaded micelles reduced capillarization, maintained the sinusoidal pores and sieve number (porosity) high. Quantitative analysis showed that CBDL caused a five to six-fold reduction in pore size and the number of fenestrae. After two weeks of treatment with MDB5 loaded micelles, fenestrations were found grouped into sieve plates (Fig. 8B), and the porosity was comparable to sham mice and significantly higher than GDC-0449 loaded micelle group. Further, in line with other results, treatment of MDB5 loaded micelles was more effective compared to GDC-0449 loaded micelles in reducing capillarization marker CD31 (Fig. 8C). Present data demonstrate that CBDL induces capillarization of liver sinusoids, and Hh pathway plays an essential role in this mechanism.

### Intrahepatic distribution of fluorescent oligo loaded micelles

Distribution of drug-loaded micelles into the fibrotic liver is likely to be reduced due to ECM deposition and sinusoidal capillarization. To evaluate whether the distribution of micelles could be restored following Hh inhibition, we encapsulated Cy7.5 labeled SCR-oligo into PEG-PCC-DC-TEPA copolymer 18. After 5 injections of GDC-0449 or MDB5 loaded micelles at the dose of 10 mg/kg for 2 weeks, mice were injected with Cy7.5-labeled micelles (2mg/kg). Liver tissues were harvested at 4 h after systemic administration of Cy7.5-labeled micelles and cryo-sectioned for examination (Fig. 8D). Sham-operated animals showed a high distribution of micelles throughout the liver, whereas CBDL animals showed significantly low distribution. Mice that received GDC-0449 or MDB5 treatment before receiving Cy7.5-labeled micelles, the signal was detected throughout the liver, although the pattern was rather heterogeneous; occasionally clusters of Cy7.5 fluorescence were found in close proximity to vessel structure or portal triads, wherein more wide fluorescence distribution was observed in the MDB5 group than the GDC-0449 group (Fig. 8E). The relative levels of fluorescence observed in the liver tissues seemed to be in a good agreement to the SEM study where relative numbers of fenestra were higher in the mouse livers of MDB5 loaded micelle treated group than GDC-0449 loaded micelle treated group. Thus, after MDB5 treatment, there was a significant increase in the delivery of fluorescent labeled SCR-oligo loaded micelles into the fibrotic liver compared to blank micelles treated mice. Further, the fluorescence signal was quantified after extracting Cy7.5 labeled oligo from the livers of different treatment groups. Figure 8F shows that prior treatment with MDB5 micelles treatment significantly increased fluorescent oligo distribution in liver compared to the liver of mice treated with GDC-0449 loaded micelles.

We further determined the effect of drug-loaded micelles on the histology of other organs like kidney and spleen. The sham mouse kidney was characterized by a clear organizational structure, with no macrophage infiltration. CBDL caused the swelling of renal tubular epithelial cells, vascular damage, necrosis, and infiltration of inflammatory cells in the kidney. There was significant decrease in structural damage and macrophage infiltration in the kidney of the mice treated with GDC-0449 loaded micelles. However, there was complete restoration of kidney histology, and almost no sign of inflammation in the mice treated with MDB5 loaded micelles (Fig. 8G, upper panel). Sham mice spleen showed the white pulp in an oval shape and consisted mainly of lymphocytes, and the red pulp consisted of red blood cells mainly, and macrophages surrounded the white pulp (Fig. 8G, lower panel). Spleen from CBDL mice showed clear structural damage, a larger white pulp (WP), and the circular region was mostly surrounded by neutrophils. The red pulp structure was restored after treatment with GDC-0449 and MDB5 loaded micelles. MDB5 loaded micelles had a more positive protective effect on spleen structural restoration than GDC-0449 loaded micelles.

## Discussion

Aberrant Hh signaling is found in most human cancers and other regenerative processes. Hh activation is usually caused by either high expression of Hh ligands or mutations in Hh pathway components (PTCH1, SMO). Hh signaling activates GLI transcription factor resulting in the activation of several downstream genes 31. Hh signaling regulates BCL2 to promote cell survival, Snail (SNAI1), and ZEB1 to promote EMT, and OPN to promote inflammation 32-34. Targeting Hh signaling components or epigenetic regulators are therefore of major therapeutic interest. Treatment with GDC-0449 and Sonidegib often show adverse effects such as weight loss, alopecia, and fatigue result in patient compliance issues 35. Therefore, novel drugs with high efficacy and lower side effects would be beneficial for a wide spectrum of the disease having high Hh activity. In our previous studies, we have shown that Hh inhibitors cyclopamine and GDC-0449 can play an important role in treating liver fibrosis by inhibiting several pro-fibrotic genes 15, 36.

Recently, we synthesized a series of new compounds and identified MDB5 as a lead candidate for SMO inhibition. MDB5 bears the N-[3-(2-pyridinyl) phenyl] benzamide (GDC-0449) scaffold. Previously we reported that due to the presence of an additional 2-pyridylmethyl group, MDB5 binds with extra interactions in 7-TM domain of Smo and thus became a stronger binder than GDC-0449 14. In this study, we evaluated anti-fibrotic efficacy of MDB5 compared to GDC-0449. Although Hh inhibitors are neither cytotoxic nor cell survival is the Hh pathway dependent, however GDC-0449 has been shown to affect cell proliferation by controlling the cell cycle 37. The activity of cyclin D is necessary for progression through a G1 phase of the cell cycle, and are sensors for mitogenic stimulation, such as Shh. Specifically, Shh increases in cyclin D1 protein levels in proliferating cells through GLI1 38. Hh pathway inhibition has been shown to increase in the percentage of cells in G1 phase with increasing concentration, in parallel cells in S phase decreased in a dose-dependent manner. We observed a similar trend when HSC-T6 cells were treated with MDB5 or GDC-0449 at their IC_50_. Both these drugs resulted in G_1_ arrest and decrease in S phase of cells, confirming the same mechanisms of action. However, MDB5 comparatively showed a significant decrease in the HSC proliferation rate at 48 h of treatment, which was dose-dependent. Marked morphological changes and increased percentage of early as well as late apoptotic cells following MDB5 treatment revealed its pro-apoptotic effect through Hh inhibition in these cells 39.

GLI1 is the main transcription factor downstream of the Hh signaling pathway 31. We found a significant up-regulation of GLI1 and PTCH1 at mRNA and protein levels in HSC-T6 cells. Elevated level of transcriptional coactivator with PDZ-binding motif YAP was recently reported in fibrosis 40. Further, knockdown of this protein results in reduced levels of pro-collagen and α-SMA 41. During fibrosis, F-actin polymerization inactivates the Hippo core kinase complex leading to the dephosphorylation of YAP and therefore its expression becomes detectable during restoration of liver mass following injury 42, 43. YAP activation controls metabolic enzyme GLS1 to enhance glutaminolysis which uses replenished aspartate for anabolic biosynthesis, which was critical for sustained proliferation and migration of HSCs within ECM 44. YAP1 is a lead mediator of Hh-directed changes in this process, and its knockdown results in reduced GLS1 gene expression 45. Furthermore, GLS1 suppression decreases MF marker α-SMA and collagen production. Notably, MDB5 treatment also significantly decreased protein levels of YAP1 and GLS1 than GDC-0449.

We previously developed PEG-PCC based polymeric micelles to enhance systemic delivery of therapeutic agents 11. PEG-PCC based micelles increase circulation time and hepatic uptake of drugs *in vivo* after intravenous administration in CBDL mice 46. In this study, we formulated MDB5 loaded micelles using the same polymer which resulted in high loading and sustained release of the drug. Importantly, blank micelles and MDB5 loaded micelles showed similar hydrodynamic size and stability over time, which suggests that the drug encapsulation has minimum effect on long-term storage of micelles. *In vitro* results obtained from cell viability, confirming that micelles were more effective with IC_50_ of ~25µM (half of the free drug) for both drugs, but there was no statistical difference among them. The discrepancy may be because of the small size of micelles, which may induce more cellular uptake and cytotoxicity as reported in the literature 47. These results were very encouraging and suggested a promising *in vivo* therapeutic outcome if such concentrations can be achieved *in vivo* following systemic administration.

The pathology resulting from CBDL is identical to human chronic cholestatic disease and characterized by initiation of a ductular reactions, and activation of portal tract fibroblasts leading to portal-portal bridging fibrosis. CBDL significantly increased serum enzymes ALT, AST, TBIL, and ALP levels compared to sham mice, which is consistent with our previous reports 15, 48. After Hh inhibition, both the drugs decreased liver injury, and thus these enzymes. Similar to cell culture results, MDB5 showed more effectiveness in reducing serum enzyme levels. Further, after measuring the liver weights and observing the morphology indicated that CBDL also significantly increased liver size, weight in proportion of total body weight, pitted surface, and pale tint. MDB5 loaded micelles treated mouse livers were closer to normal liver in appearance. Liver sections from control and treated animals were stained with H&E. CBDL animals showed cell injury and necrosis in the periportal regions accompanied with marked fibrous connective tissue.

Hh stimulated myofibroblasts are responsible for the production of a collagen matrix in CBDL mice 49. Treatment with MDB5 loaded micelles significantly decreased collagen deposition and hydroxyproline content (a marker of collagen deposition) in the liver section show more efficient Hh pathway inhibition than GDC-0449 loaded micelles.

As discussed earlier, the Hh pathway activated YAP in HSCs has a direct relation with metabolic enzyme GLS1, and glutaminolysis 50. We observed significant downregulation of GLS1 protein by immunostaining *in vivo* by MDB5. During liver fibrosis progression, various liver cell types such as hepatocytes and cholangiocytes start proliferation. Expression of cell proliferating marker Ki-67 increased after CBDL, suggesting these cells undergo mitosis in response to injury 51. This proliferative response was reversed in MDB5 treated mice within 2 weeks comparable to the sham. Osteopontin (OPN) expression is low in normal liver, while it upregulates during stress, cell injury, and pathology. OPN plays an important role particularly in the regulation of inflammation, tissue remodeling, and cell survival 52. Protein OPN has an integrin-binding arginine-glycine-aspartate (RGD) sequence which can interact with receptors α_v_(β_1_, β_3_, or β_5_) and (α_4_, α_5_, α_8_, or α_9_)β_1_, and can deliver an antiapoptotic ECM-like signal to surrounding cells 53.

We also observed OPN was negligible in the sham liver, and heavily expressed by biliary epithelial cells, and the necrotic area in CBDL liver. After treatment with GDC-0449 and MDB5, OPN was decreased showing less count of inflammatory cells and necrosis. In the absence of Hh ligands or any other stimuli, Smo is inactive, and Gli2 is degraded in the cytoplasm. While after Hh activation, Gli2 increase transcription of its target gene Gli1 54. GLI proteins (Gli1/2/3) are a Kruppel-like family of transcription factors with a zinc-finger DNA-binding domain 31. When SMO is active, Gli2 predominately induces Gli1 expression, which acts as a transcriptional activator and regulates the expression of Hh target genes. As a result of CBDL, we observed that GLI1 and PTCH1 expression were high in the liver at both mRNA and protein levels. MDB5 showed better efficacy and decreased their expression after treatment compared to GDC-0449. When we co-stained slides with GLI1 and GLS1, co-expression was mainly by cells of the perivascular region and cholangiocytes. While GDC-0449 decreased these protein staining, by cholangiocytes, but not eliminated by cells in the bile duct surrounding area, MDB5 treated sample showed minimum localization, instead GLS1 expression was still observable.

Myofibroblasts are pro-fibrogenic cells located in peri-sinusoidal and centrilobular region and express the marker α-SMA. Recent studies suggest that Gli1^+^ cells after injury differentiate into myofibroblasts in organ fibrosis and acquire the key features of myofibroblasts including α-SMA expression 29. We observed minimum α-SMA positive cells surrounding portal arterioles. We observed Gli1^+^ cells proliferate and differentiate in sham mice into myofibroblasts in CBDL mice. Mice treated with GDC-0449 loaded micelles remained significantly more periportal α-SMA positive cells than mice treated with MDB5 loaded micelles. Cadherins mediate cell to cell adhesion, and liver fibrosis is accompanied by the loss of E-cadherin (ECAD) by myofibroblasts while the process of EMT 55. ECAD antagonize the profibrogenic TGF-β1 pathway by decreasing Smad3/2 phosphorylation, whereas loss of ECAD promotes the up-regulation of TGF-*β*1 and its target genes and facilitates liver fibrosis. Forced expression of ECAD is known to downregulate α-SMA expression in HSCs 55. Similar to periportal α-SMA staining, significant increase in the number of ECAD positive cells seen in mice after CBDL, which was reversible after MDB5 treatment. Hh responsive Gli1^+^ fibroblasts are mainly α-SMA expressing cells in fibrosis, therefore we observed localization of GLI1 and α-SMA in myofibroblasts which is supported by previous reports 56.

The liver sinusoidal endothelial cells (LSEC) have a unique phenotype among all mammalian endothelial cells, i.e., LSEC has open fenestrae grouped into sieve plates and lack an organized basement membrane. LSEC lose this highly specialized morphology during a process called capillarization. LSEC are Hh responsive and express Hh-target genes such as Gli1/2, PTCH, soluble frizzled related peptide 1 (sFRP1), OPN, and Twist post liver injury. Inhibition of Hh signaling also repressed expression of capillarization-associated genes, such as inducible nitric oxide synthase (iNOS), CD31 and endothelin 1 (ET-1). Immunostaining for CD31 a marker of capillarised LSECs was evident in our CBDL liver sample 57. Several drugs are known to dilate fenestrae, such as pantethine, acetylcholine, and ethanol; whereas drugs such as nicotine, long-term ethanol abuse, adrenalin, noradrenalin, and serotonin decrease the endothelial porosity. The disappearance of the normal filtration barrier in cirrhotic livers limits the exchange between the sinusoidal blood and the parenchymal cells and is a major contributor to hepatic failure in cirrhosis 58. The defenestration precedes hepatic fibrosis and only reversible before the establishment of the endothelial basement membrane, and cirrhosis 59. This is very intriguing since de-differentiated (non capillarised) LSEC also antagonize HSC activation by directly acting on the HSC themselves to prevent/reverse the myofibroblastic phenotype or by VEGF upregulation 5. Hh signaling induces Neuropilin expression resulting LSEC migration and tube formation. Inhibition of Hh signaling by GDC-0449 and MDB5 promoted restitution of fenestrated LSEC.

We previously reported decrease in intrahepatic distribution of micelles after CBDL. Capillarization could be a possible reason for this observation. In general, endothelial fenestrae measure 50-300 nm in diameter in normal human liver, and the grouped fenestrae act as a dynamic filter 60. The filtration of our micelles (size range 50 ± 10 nm) by open pores is the simplest mechanism for liver entrance, and we observed a high fluorescent signal in sham mice after 4 h of administration of Cy7.5 labeled oligo loaded micelles. A reasonable decrease accumulation of fluorescence was seen in the CBDL mice due to capillarization. Treatment with MDB5 and GDC-0449 restored micelles filtration and showed higher fluorescence than CBDL, wherein MDB5 was more effective than GDC-0449.

Hepatorenal syndrome (HRS) is a condition of renal failure in patients with the advanced liver disease without any identifiable renal pathology. Portal hypertension developed in liver fibrosis disturbs systemic and renal hemodynamics 61. Histological examination of kidney tissues showed normal glomerular and tubular histology in the sham group, however, kidney tissues after CBDL showed slightly swelling and necrosis of tubular epithelial cells which is in line with literature 62. Strikingly, all these pathological features were restored in GDC-0449 treated group, and completely disappeared in MDB5 treatment groups, suggesting a protective effect not only on the liver but kidney as well. Liver cirrhosis is frequently accompanied by splenomegaly and often attributed to hypersplenism (functional overactivity of the spleen) 63, leading to decrease in platelet and hemoglobin levels. The white pulp area which represents the lymphoid tissue of spleen tended to be larger in the spleens of CBDL group than that of the sham group. Thus, it might be concluded that an overall increase in the absolute splenic lymphoid tissue contributes to the enlargement of the spleen in liver fibrosis 64. After 2-weeks of treatment, GDC-0449 and MBD5 markedly attenuated the enlargement of splenic lymphoid tissue compared with CBDL animals.

## Conclusions

Compared to GDC-0449, we have shown higher Hh pathway inhibition *in vitro* and *in vivo* by MDB5. This conclusion is based on its higher potency in suppression of Gli1 signaling, and the blockade of Hh driven other cellular pathways. Further, formulation development was successfully completed for MDB5. A major deficiency of our study is the lack of pharmacokinetics and biodistribution study of MDB5 to predict its future in clinics. Collectively, the current inhibitor may present an opportunity for medicinal chemistry optimization on pharmaceutical property towards the development of new liver fibrotic treatment.

## Supplementary Material

Supplementary figures and tables.

## References

[1] Kumar V, Mahato RI (2015). Delivery and targeting of miRNAs for treating liver fibrosis. Pharm Res.

[2] Chang H, Meng HY, Liu SM (2017). Identification of key metabolic changes during liver fibrosis progression in rats using a urine and serum metabolomics approach. Sci Rep.

[3] Mullor JL, Sanchez P, Ruiz i Altaba A (2002). Pathways and consequences: Hedgehog signaling in human disease. Trends Cell Biol.

[4] Taipale J, Beachy PA (2001). The hedgehog and wnt signalling pathways in cancer. Nature.

[5] Xie G, Choi SS, Syn WK (2013). Hedgehog signalling regulates liver sinusoidal endothelial cell capillarisation. Gut.

[6] Pospisilik JA, Schramek D, Schnidar H (2010). Drosophila genome-wide obesity screen reveals hedgehog as a determinant of brown versus white adipose cell fate. Cell.

[7] Swiderska-Syn M, Xie G, Michelotti GA (2016). Hedgehog regulates yes-associated protein 1 in regenerating mouse liver. Hepatology.

[8] Shi C, Cai Y, Li Y (2018). Yap promotes hepatocellular carcinoma metastasis and mobilization via governing cofilin/F-actin/lamellipodium axis by regulation of JNK/Bnip3/SERCA/CaMKII pathways. Redox Biol.

[9] Poisson J, Lemoinne S, Boulanger C (2017). Liver sinusoidal endothelial cells: Physiology and role in liver diseases. J Hepatol.

[10] Warren A, Bertolino P, Benseler V (2007). Marked changes of the hepatic sinusoid in a transgenic mouse model of acute immune-mediated hepatitis. J Hepatol.

[11] Kumar V, Mondal G, Slavik P (2015). Codelivery of small molecule hedgehog inhibitor and miRNA for treating pancreatic cancer. Mol Pharm.

[12] Gruber W, Peer E, Elmer DP (2018). Targeting class I histone deacetylases by the novel small molecule inhibitor 4SC-202 blocks oncogenic hedgehog-GLI signaling and overcomes smoothened inhibitor resistance. Int J Cancer.

[13] Kumar V, Mundra V, Mahato RI (2014). Nanomedicines of hedgehog inhibitor and PPAR-gamma agonist for treating liver fibrosis. Pharm Res.

[14] Kumar V, Chaudhary AK, Dong Y (2017). Design, synthesis and biological evaluation of novel hedgehog inhibitors for treating pancreatic cancer. Sci Rep.

[15] Kumar V, Mondal G, Dutta R (2016). Co-delivery of small molecule hedgehog inhibitor and miRNA for treating liver fibrosis. Biomaterials.

[16] Zbyszynski P, Tomasini-Johansson BR, Peters DM (2018). Characterization of the PEGylated functional upstream domain peptide (PEG-FUD): A potent fibronectin assembly inhibitor with potential as an anti-fibrotic therapeutic. Pharm Res.

[17] Kattel K, Mondal G, Lin F (2017). Biodistribution of self-assembling polymer-gemcitabine conjugate after systemic administration into orthotopic pancreatic tumor bearing mice. Mol Pharm.

[18] Kumar V, Mundra V, Peng Y (2018). Pharmacokinetics and biodistribution of polymeric micelles containing miRNA and small-molecule drug in orthotopic pancreatic tumor-bearing mice. Theranostics.

[19] Li Y, Pu S, Liu Q (2019). An integrin-based nanoparticle that targets activated hepatic stellate cells and alleviates liver fibrosis. J Control Release.

[20] Gajendiran P, Vega LI, Itoh K (2018). Elevated mitochondrial activity distinguishes fibrogenic hepatic stellate cells and sensitizes for selective inhibition by mitotropic doxorubicin. J Cell Mol Med.

[21] Singh P, Carraher C, Schwarzbauer JE (2010). Assembly of fibronectin extracellular matrix. Annu Rev Cell Dev Biol.

[22] Sakar MS, Eyckmans J, Pieters R (2016). Cellular forces and matrix assembly coordinate fibrous tissue repair. Nat Commun.

[23] Wells RG (2008). Cellular sources of extracellular matrix in hepatic fibrosis. Clin Liver Dis.

[24] Jin L, Alesi GN, Kang S (2016). Glutaminolysis as a target for cancer therapy. Oncogene.

[25] Li J, Ghazwani M, Liu K (2017). Regulation of hepatic stellate cell proliferation and activation by glutamine metabolism. PLoS One.

[26] Crespo Yanguas S, da Silva TC, Pereira IVA (2018). Genetic ablation of pannexin1 counteracts liver fibrosis in a chemical, but not in a surgical mouse model. Arch Toxicol.

[27] Syn WK, Choi SS, Liaskou E (2011). Osteopontin is induced by hedgehog pathway activation and promotes fibrosis progression in nonalcoholic steatohepatitis. Hepatology.

[28] Yu F, Geng W, Dong P (2018). LncRNA-MEG3 inhibits activation of hepatic stellate cells through SMO protein and miR-212. Cell Death Dis.

[29] Kramann R, Schneider RK, DiRocco DP (2015). Perivascular Gli1+ progenitors are key contributors to injury-induced organ fibrosis. Cell Stem Cell.

[30] Bansal R, Nakagawa S, Yazdani S (2017). Integrin alpha 11 in the regulation of the myofibroblast phenotype: Implications for fibrotic diseases. Exp Mol Med.

[31] Bariwal J, Kumar V, Dong Y (2018). Design of hedgehog pathway inhibitors for cancer treatment.

[32] Song M, Ou X, Xiao C (2013). Hedgehog signaling inhibitor cyclopamine induces apoptosis by decreasing Gli2 and Bcl2 expression in human salivary pleomorphic adenoma cells. Biomed Rep.

[33] Katoh Y, Katoh M (2009). Hedgehog target genes: Mechanisms of carcinogenesis induced by aberrant hedgehog signaling activation. Curr Mol Med.

[34] Denhardt DT, Giachelli CM, Rittling SR (2001). Role of osteopontin in cellular signaling and toxicant injury. Annu Rev Pharmacol Toxicol.

[35] Dessinioti C, Antoniou C, Stratigos AJ (2017). From basal cell carcinoma morphogenesis to the alopecia induced by hedgehog inhibitors: Connecting the dots. Br J Dermatol.

[36] Pratap A, Panakanti R, Yang N (2011). Cyclopamine attenuates acute warm ischemia reperfusion injury in cholestatic rat liver: Hope for marginal livers. Mol Pharm.

[37] Wu C, Hu S, Cheng J (2017). Smoothened antagonist GDC-0449 (vismodegib) inhibits proliferation and triggers apoptosis in colon cancer cell lines. Exp Ther Med.

[38] Choi SS, Omenetti A, Witek RP (2009). Hedgehog pathway activation and epithelial-to-mesenchymal transitions during myofibroblastic transformation of rat hepatic cells in culture and cirrhosis. Am J Physiol Gastrointest Liver Physiol.

[39] Lin Z, Sheng H, You C (2017). Inhibition of the CyclinD1 promoter in response to sonic hedgehog signaling pathway transduction is mediated by Gli1. Exp Ther Med.

[40] Liu F, Lagares D, Choi KM (2015). Mechanosignaling through YAP and TAZ drives fibroblast activation and fibrosis. Am J Physiol Lung Cell Mol Physiol.

[41] Mannaerts I, Leite SB, Verhulst S (2015). The hippo pathway effector YAP controls mouse hepatic stellate cell activation. J Hepatol.

[42] Piersma B, Bank RA, Boersema M (2015). Signaling in fibrosis: TGF-beta, WNT, and YAP/TAZ converge. Front Med (Lausanne).

[43] Lu L, Finegold MJ, Johnson RL (2018). Hippo pathway coactivators yap and taz are required to coordinate mammalian liver regeneration. Exp Mol Med.

[44] Bertero T, Oldham WM, Cottrill KA (2016). Vascular stiffness mechanoactivates YAP/TAZ-dependent glutaminolysis to drive pulmonary hypertension. J Clin Invest.

[45] Zhang X, Zhao H, Li Y (2018). The role of YAP/TAZ activity in cancer metabolic reprogramming. Mol Cancer.

[46] Dutta R, Kumar V, Peng Y (2017). Pharmacokinetics and biodistribution of GDC-0449 loaded micelles in normal and liver fibrotic mice. Pharm Res.

[47] Wang Y, Liang X, Tong R (2018). Gambogic acid-loaded polymeric micelles for improved therapeutic effect in breast cancer. J Biomed Nanotechnol.

[48] Kumar V, Kumar V, Luo J (2018). Therapeutic potential of OMe-PS-miR-29b1 for treating liver fibrosis. Mol Ther.

[49] Omenetti A, Popov Y, Jung Y (2008). The hedgehog pathway regulates remodelling responses to biliary obstruction in rats. Gut.

[50] Du K, Hyun J, Premont RT (2018). Hedgehog-YAP signaling pathway regulates glutaminolysis to control activation of hepatic stellate cells. Gastroenterology.

[51] Abshagen K, Konig M, Hoppe A (2015). Pathobiochemical signatures of cholestatic liver disease in bile duct ligated mice. BMC Syst Biol.

[52] Lorena D, Darby IA, Gadeau AP (2006). Osteopontin expression in normal and fibrotic liver. altered liver healing in osteopontin-deficient mice. J Hepatol.

[53] Denhardt DT, Noda M, O'Regan AW (2001). Osteopontin as a means to cope with environmental insults: Regulation of inflammation, tissue remodeling, and cell survival. J Clin Invest.

[54] Ikram MS, Neill GW, Regl G (2004). GLI2 is expressed in normal human epidermis and BCC and induces GLI1 expression by binding to its promoter. J Invest Dermatol.

[55] Cho IJ, Kim YW, Han CY (2010). E-cadherin antagonizes transforming growth factor beta1 gene induction in hepatic stellate cells by inhibiting RhoA-dependent Smad3 phosphorylation. Hepatology.

[56] Moshai EF, Wemeau-Stervinou L, Cigna N (2014). Targeting the hedgehog-glioma-associated oncogene homolog pathway inhibits bleomycin-induced lung fibrosis in mice. Am J Respir Cell Mol Biol.

[57] Xing Y, Zhao T, Gao X (2016). Liver X receptor alpha is essential for the capillarization of liver sinusoidal endothelial cells in liver injury. Sci Rep.

[58] Fraser R, Dobbs BR, Rogers GW (1995). Lipoproteins and the liver sieve: The role of the fenestrated sinusoidal endothelium in lipoprotein metabolism, atherosclerosis, and cirrhosis. Hepatology.

[59] Braet F, Wisse E (2002). Structural and functional aspects of liver sinusoidal endothelial cell fenestrae: A review. Comp Hepatol.

[60] Romero EL, Morilla MJ, Regts J (1999). On the mechanism of hepatic transendothelial passage of large liposomes. FEBS Lett.

[61] Yeung E, Yong E, Wong F (2004). Renal dysfunction in cirrhosis: Diagnosis, treatment and prevention. MedGenMed.

[62] Fickert P, Krones E, Pollheimer MJ (2013). Bile acids trigger cholemic nephropathy in common bile-duct-ligated mice. Hepatology.

[63] Li L, Duan M, Chen W (2017). The spleen in liver cirrhosis: Revisiting an old enemy with novel targets. J Transl Med.

[64] Chen Y, Wang W, Wang H (2016). Rapamycin attenuates splenomegaly in both intrahepatic and prehepatic portal hypertensive rats by blocking mTOR signaling pathway. PLoS One.

