# Supplementary material for: The use of micelles to deliver potential hedgehog pathway inhibitor for the treatment of liver fibrosis
Source: Theranostics. 2019 Oct 12;9(25):7537–55. doi: 10.7150/thno.38913 (PMC6831471; doi:10.7150/thno.38913)
Supplement: Supplementary file 1 — Supplementary figures and tables. [file thnov09p7537s1.pdf]

Supplementary Figure S1

A

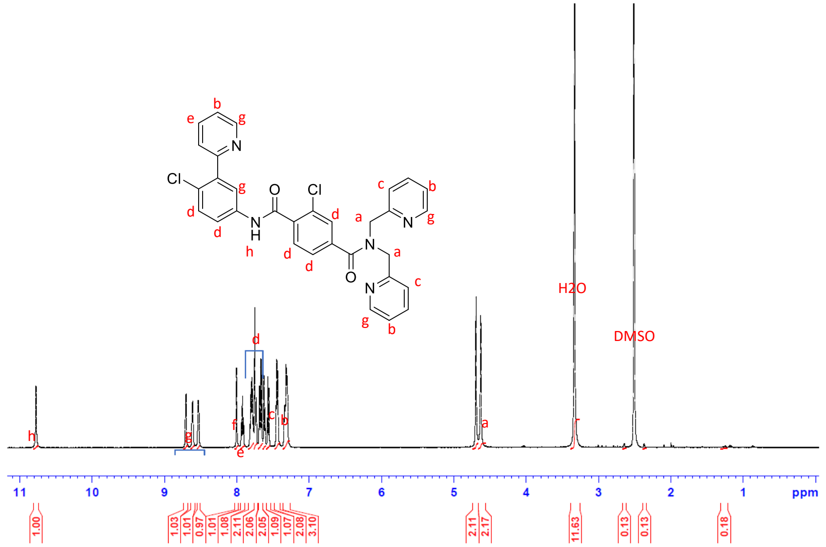

B

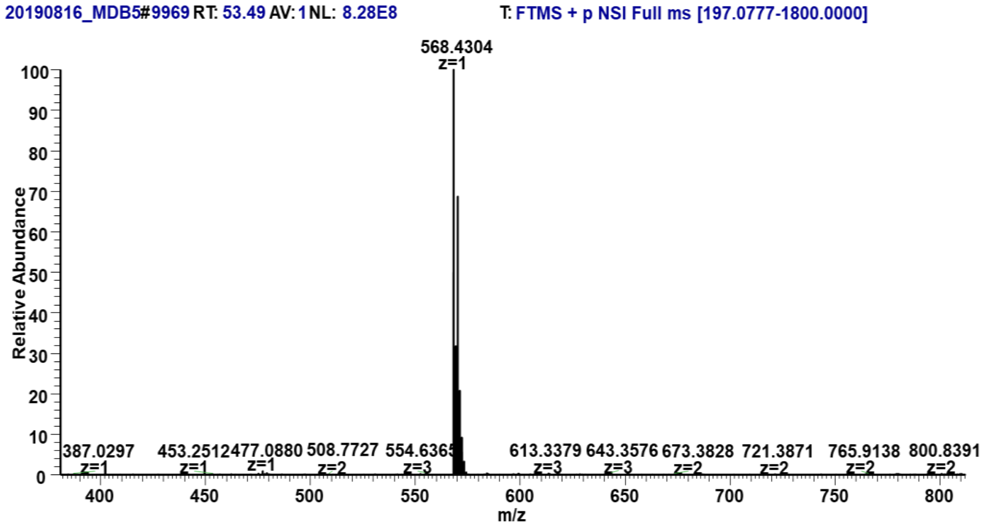

C

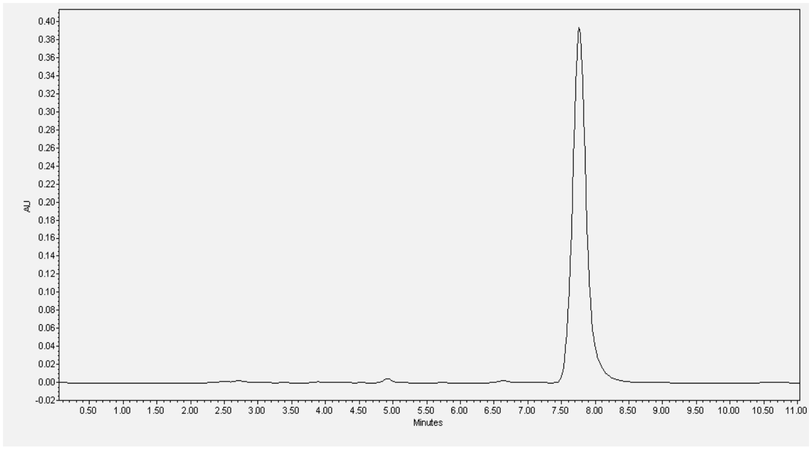

Figure S1. Characterization of MDB5 molecule. (A) Proton NMR, **(B)** Mass spectrum of MDB5. **(C)** HPLC Chromatogram.

Supplementary Figure S2

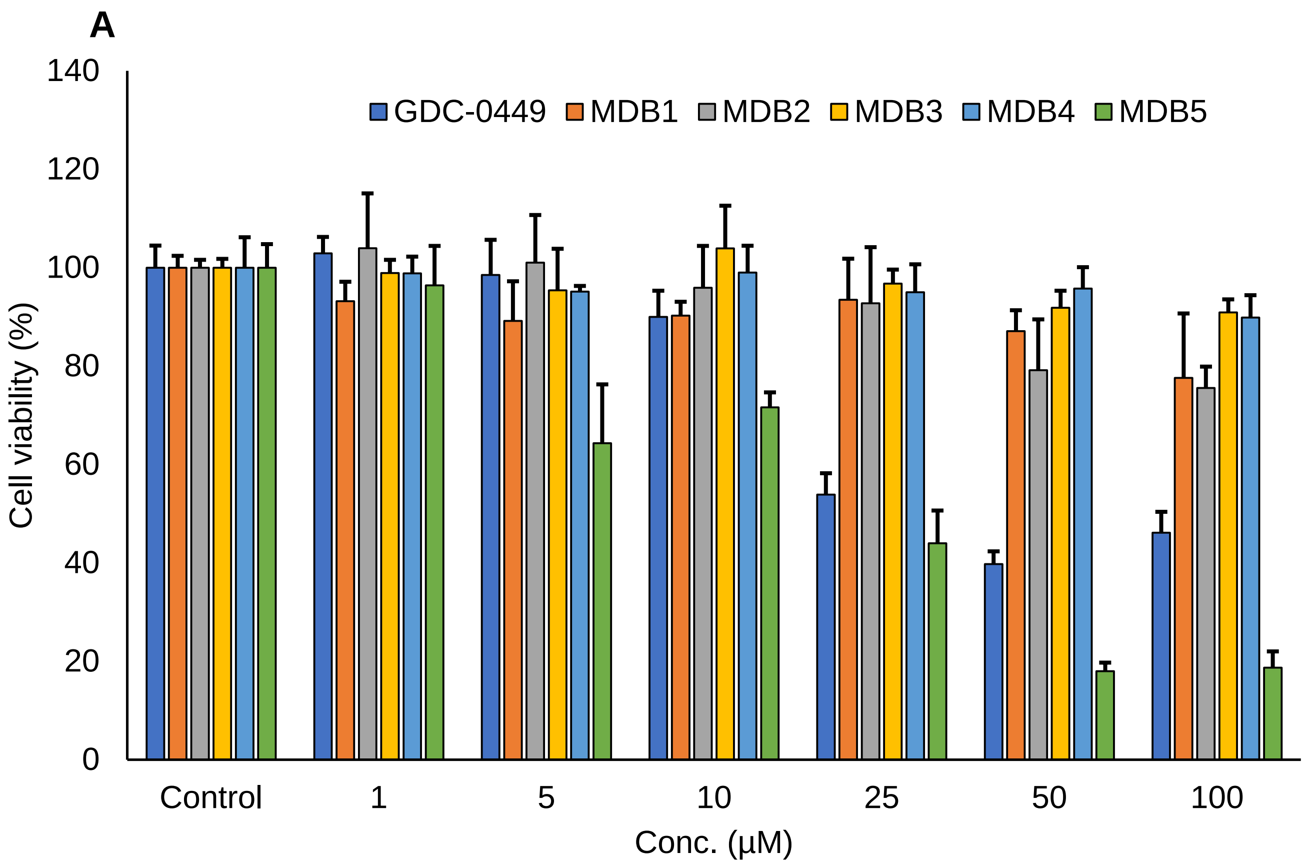

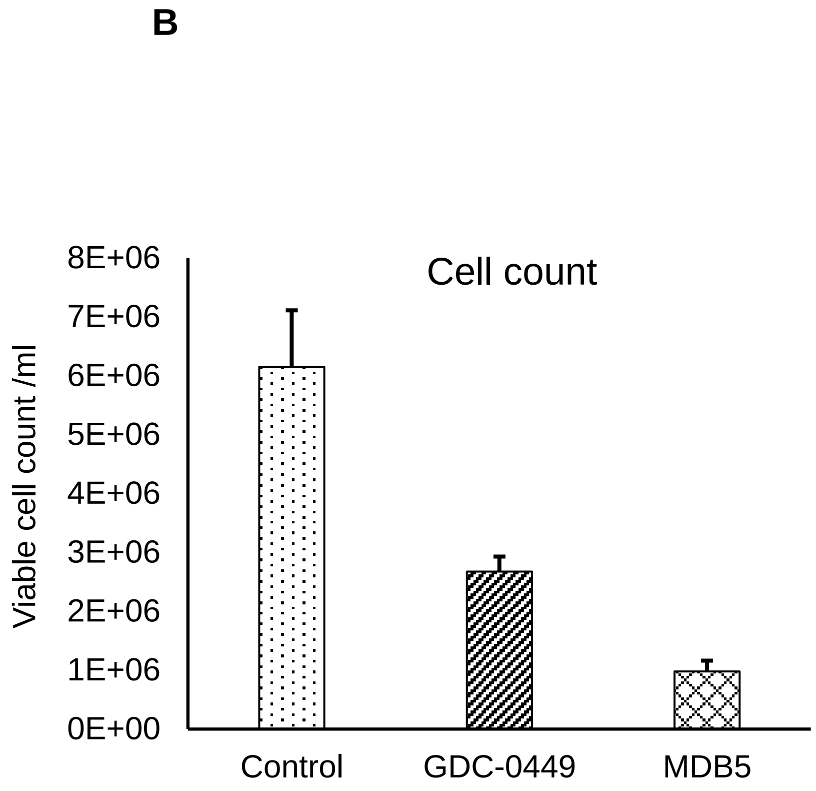

Figure S2. (A) Cell viability with different GDC-0449 analogs. **(B)** viable cell count after GDC-0449 and MDB5 treatment.

Supplementary Figure S3

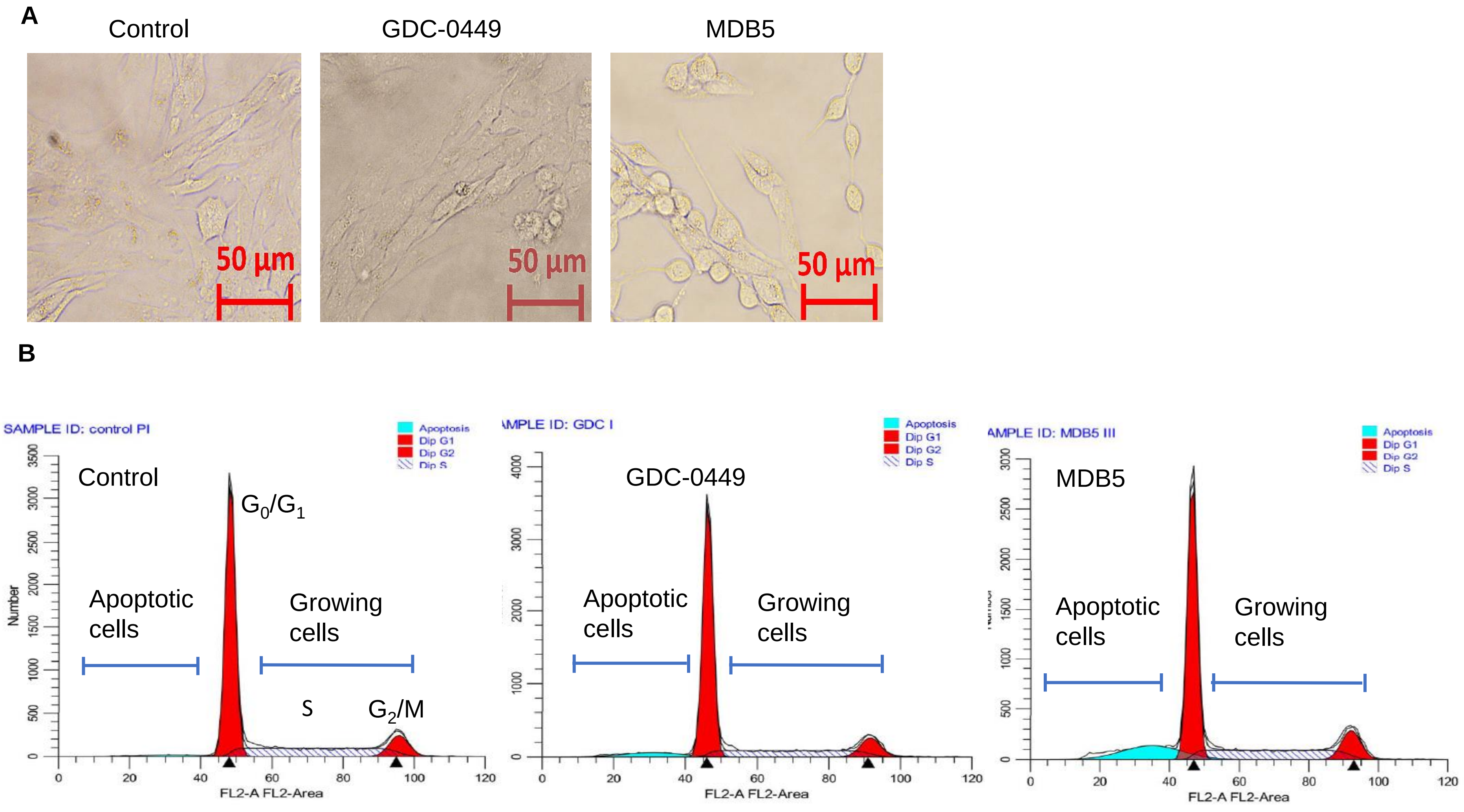

Figure S3. (A) Cell cycle analysis and **(B)** cell morphology after GDC-0449 and MDB5 treatment.

Supplementary Figure S4

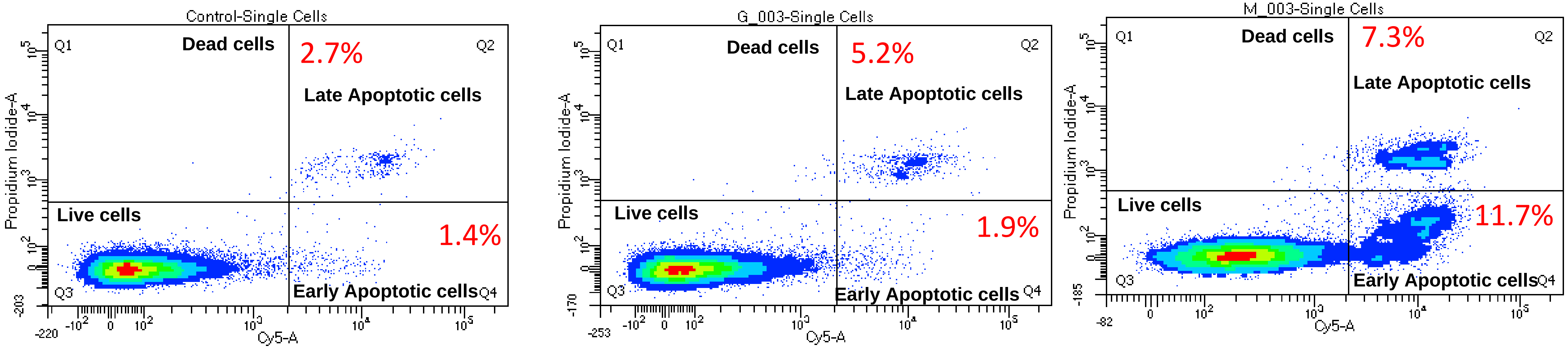

Figure S4. % of apoptosis after GDC-0449 and MDB5 treatment.

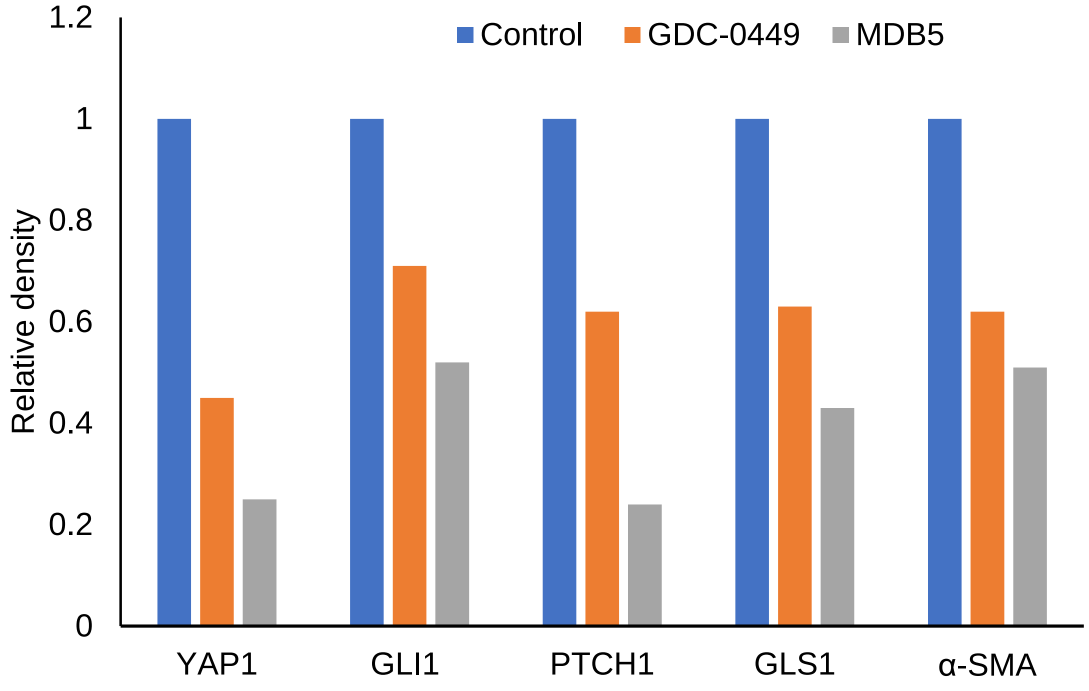

Figure S5. Quantification (relative density) of the intensity of staining of YAP1, GLI1, PTCH1, GLS1, and α-SMA protein detected by Western blot.
